# PerSVade: personalized structural variant detection in any species of interest

**DOI:** 10.1186/s13059-022-02737-4

**Published:** 2022-08-16

**Authors:** Miquel Àngel Schikora-Tamarit, Toni Gabaldón

**Affiliations:** 1 Barcelona Supercomputing Centre (BSC-CNS), Plaça Eusebi Güell, 1-3, 08034 Barcelona, Spain; 2 Institute for Research in Biomedicine (IRB Barcelona), The Barcelona Institute of Science and Technology, Baldiri Reixac, 10, 08028 Barcelona, Spain; 3 Catalan Institution for Research and Advanced Studies (ICREA), Barcelona, Spain; 4 Centro Investigación Biomédica En Red de Enfermedades Infecciosas, Barcelona, Spain

**Keywords:** Structural variants, Variant calling, Short reads, Parameter optimization

## Abstract

Structural variants (SVs) underlie genomic variation but are often overlooked due to difficult detection from short reads. Most algorithms have been tested on humans, and it remains unclear how applicable they are in other organisms. To solve this, we develop perSVade (personalized structural variation detection), a sample-tailored pipeline that provides optimally called SVs and their inferred accuracy, as well as small and copy number variants. PerSVade increases SV calling accuracy on a benchmark of six eukaryotes. We find no universal set of optimal parameters, underscoring the need for sample-specific parameter optimization. PerSVade will facilitate SV detection and study across diverse organisms.

**Supplementary Information:**

The online version contains supplementary material available at 10.1186/s13059-022-02737-4.

## Background

Structural variants (SVs) are large changes (typically >50 bp) in the DNA between individuals that alter genome size (duplications and deletions) or generate rearrangements (inversions, translocations, and interspersed insertions) [1, 2]. In eukaryotes, SVs can drive clinically relevant phenotypes including cancer [3–5], neurological diseases [6, 7], or antifungal drug resistance [8, 9]. In addition, SVs may generate significant intraspecific genetic variation across many taxa like humans [10–12], songbirds [13], or rice plants [14]. Despite their role on human health and natural diversity, most genomic studies overlook SVs due to technical difficulties in calling SVs from short reads [15]. This means that the role of SVs remains largely unexplored across eukaryotes.

Inferring SVs from short reads is challenging because it relies mostly on indirect evidence coming from de novo assembly alignment, changes in read depth, or the presence of discordantly paired / split reads in read mapping analysis [16–21]. Long-read-based SV calling may avoid some of these limitations, but short read-based SV calling remains a cost-effective strategy to find SVs in large cohorts [14, 15, 22]. Recent benchmarking studies compared the performance of different tools in human genomes and found that SV calling accuracy is highly dependent on the methods and filtering strategy used [15, 23, 24]. Such studies are useful to define “best practices” (optimal methods and filtering strategies) for SV calling in human samples. However, few studies have investigated the accuracy of these tools on non-human genomes. It is unclear whether the human-derived “best practices” for SV calling can be reliably used in other species. We hypothesize that this may not be the case for genomes with different contents of repetitive or transposable elements, which constrain the short read-based SV calling accuracy [24]. In summary, current tools for short-read-based SV calling are often unprepared for non-human genomes, which hinders the study of SVs in most organisms.

To overcome this limitation, we developed the *personalized structural variation detection* pipeline, or perSVade (pronounced “persuade”), which is designed to adapt a state-of-the-art SV calling pipeline to any sample/individual of any genome/species of interest. PerSVade detects breakpoints (two joined regions that exist in the sample of interest and not in the reference genome) from short paired-end reads and summarizes them into complex SVs (deletions, inversions, tandem duplications, translocations, and interspersed insertions). The pipeline provides automated benchmarking and parameter selection for these methods in any genome or sequencing run, which is useful for species without such recommended parameters. PerSVade provides an automated report of the SV calling accuracy on these simulations, which serves to estimate the confidence of the results on real samples. Beyond SV detection, perSVade can be used to find small variants (single-nucleotide polymorphisms (SNPs) and insertions/deletions (IN/DELs)) and read depth-based copy number variation (CNV), all implemented within a flexible and modular framework.

The following sections describe perSVade and its SV calling performance on various datasets of both simulated and real genomes with SVs.

## Results

### PerSVade: a pipeline to call and interpret structural variants in your species of interest

**Fig. 1:**
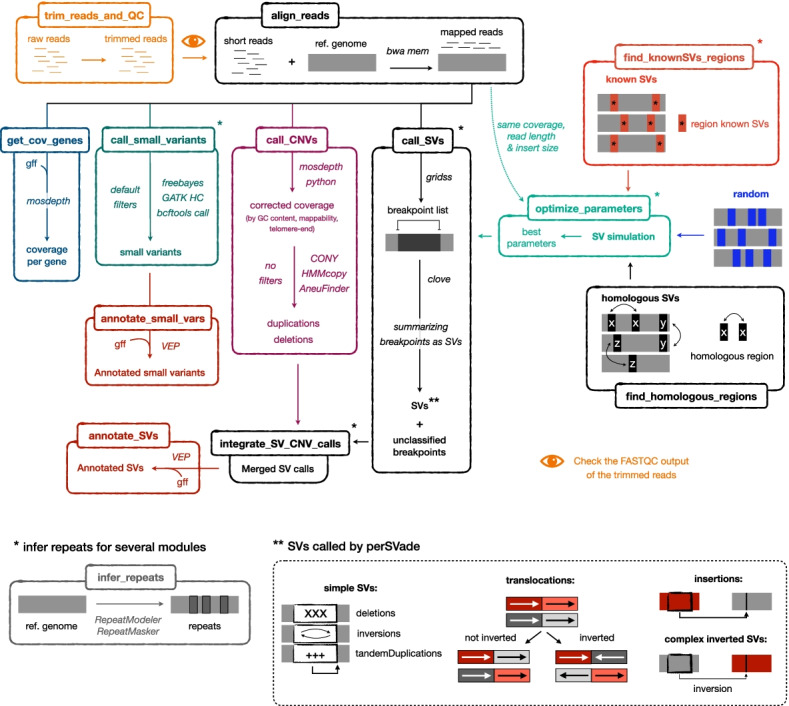
Schematic representation of the modular workflow of PerSVade. This figure shows the modules of perSVade (each represented in a different box and executable with a single command), which may be combined following the drawn arrows. The italic text describes the algorithms used at each step. The pipeline identifies either structural variants (SVs) (module “call_SVs”), coverage-derived copy number variants (CNVs) (module “call_CNVs”), small variants (module “call_small_variants”), and/or changes in the coverage per gene (module “get_cov_genes”) from aligned short paired-end reads (obtained with the module “align_reads”). The different types of SVs output by “call_SVs” are drawn at the bottom for clarity. In addition, the module “trim_treads_and_QC” can be used to trim the reads and perform quality control with *FASTQC* before read alignment*.* On another note, several modules (“call_SVs,” “find_knownSVs_regions,” “integrate_SV_CNV_calls,” “optimize_parameters,” and “call_small_variants”) use an annotation of genomic repeats that can be obtained with the module “infer_repeats” (bottom left). The most novel aspect of perSVade is the automatic parameter optimization for SV calling adapted to the input (implemented in the module “optimize_parameters”). This is achieved through simulations of SVs on the reference genome, which can be randomly placed (“random”), around regions with previously known SVs (“known”) or on regions with pairwise homology (“homologous”). The modules “find_knownSVs_regions” and “find_homologous_regions” can be used to infer these “known” and “homologous” regions, respectively. In addition, the variants found with “call_SVs” and “call_CNVs” can be combined with the module “integrate_SV_CNV_calls.” Finally, the modules “annotate_SVs” and “annotate_small_vars” can be used to obtain a functional annotation of the variants. See “Methods” for more details. In addition, note that Additional file 1: Figure S1 includes a more detailed representation of how “optimize_parameters” works

PerSVade identifies SVs from a paired-end WGS dataset and a reference genome as sole inputs. It identifies breakpoints from the aligned reads with *gridss* [21] and summarizes them into actual SVs (insertions, translocations, deletions, inversion, and tandem duplications) with *clove* [25]. We followed the recent recommendation of using a single, high-performing algorithm for breakpoint calling instead of using multiple software [24]. We chose *gridss* because of its high accuracy in several benchmarking studies [23, 24]. In addition, our pipeline generates a functional annotation of the variants, which is useful to evaluate the altered genomic regions and aid downstream analyses. In summary, perSVade is a pipeline to find and interpret SVs from most eukaryotic sequencing datasets (Fig. 1).

A key feature of perSVade is the parameter optimization step (implemented in the “optimize_parameters” module and shown in Additional file 1: Figure S1). There are no specific recommendations for filtering the outputs of *gridss* and *clove* in most species, and it is unclear whether the parameters validated on model organisms are universal. Similarly, the performance of these algorithms on different sequencing formats (i.e., varying read lengths, coverage, or insert size) is not easy to predict. To solve this automatically, perSVade “optimize_parameters” generates simulated genomes (based on the reference genome and input dataset) with SVs and chooses the most accurate filters (with the highest harmonic mean between precision and recall (*F*-value)) for these simulations. To account for different mechanisms of SV formation, the simulations can be either (1) randomly placed across the genome (“random” simulations), (2) around regions with previously known SVs (“known” simulations), or (3) around regions with homologous sequences (“homologous” simulations). We consider that “known” and “homologous” simulations are more realistic than the “random” ones. See “Methods” for further details. Regardless of the simulation type, the optimized filters can be used for the SV calling on real data, potentially yielding the highest possible performance. The accuracy of the optimized filters on different simulations is reported as a tabular file, which is useful to define the expected calling accuracy. We hypothesize that this accuracy may vary across species and/or sequencing formats, and perSVade can infer it on any input sample. All in all, perSVade automatically finds the best filters and reports the expected calling accuracy for each input sample.

We validated the usability of perSVade by running it on available sequences for six phylogenetically diverse eukaryotes with different genome sizes (*Candida glabrata* (12 Mb), *Candida albicans* (14 Mb), *Cryptococcus neoformans* (19 Mb), *Arabidopsis thaliana* (120 Mb), *Drosophila melanogaster* (144 Mb), and *Homo sapiens* (3163 Mb)), with three WGS runs per species (yielding datasets with 6.75×10^6^–1.59×10^9^ reads, see “Methods”). We ran the pipeline using parameter optimization with “random,” “known,” or “homologous” simulations. In addition, we ran perSVade with default parameters as a baseline, useful to evaluate the impact of parameter optimization (the core and most novel feature of perSVade) on calling accuracy and resource consumption. We found that the computational burden (running time and memory used) was highly variable among datasets and correlated with genome and dataset sizes. As expected, parameter optimization increased resource consumption in all cases. This burden was particularly high for the human datasets, which may hinder the usage of perSVade on such large genomes if computational resources are limited (Additional file 1: Figure S2). However, we consider that such choices should be left to the user based on these results, since the increased accuracy due to parameter optimization may outweigh resource costs. Taken together, our analysis indicates that perSVade can be used for SV calling in a wide range of eukaryotes and sequencing datasets.

### PerSVade’s parameter optimization improves calling accuracy in simulated datasets

**Fig. 2:**
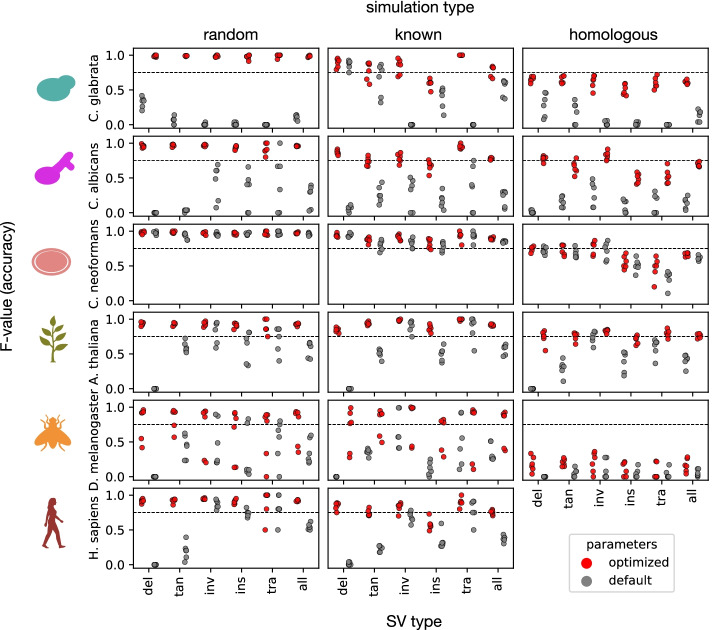
PerSVade’s parameter optimization improves the SV calling accuracy on simulations. We ran perSVade’s SV calling on three samples / species for six eukaryotes (see “Methods”) using either “random,” “known,” or “homologous” simulations. These plots show the *F*-value of either default (gray) or optimized (red) parameters (for each sample and simulation type) on these simulations. The *x* axis represents the type of SV (deletions (del), tandem duplications (tan), inversions (inv), insertions (ins), translocations (tra), and the average of all SVs (all)). Note that Additional file 1: Figure S3 shows the corresponding precision and recall, from which the *F*-value is calculated

In order to clarify the impact of parameter optimization on calling accuracy, we measured the performance of perSVade’s SV calling on these samples and simulations. We found that the *F*-value after parameter optimization on “random” and “known” simulations was high (between 0.75 and 1.0) in most samples and SV types (with one exception in *Drosophila melanogaster* that yielded an *F*-value ~ 0.5). The *F*-value on “homologous” simulations was often lower (depending on the species), suggesting that SVs happening on regions with pairwise homology may be more difficult to resolve. As expected, the accuracy on “random” SVs was higher than on more realistic simulations (“known” and “homologous”), suggesting that it may overestimate real data accuracy. In general, the *F*-value was higher than the “default” setting in most species (except in *C. neoformans*), and the improvement was dramatic in some SV types (i.e., the *F*-value went from <0.1 to >0.95 in *C. glabrata*’s deletions or insertions) (Fig. 2). In addition, we found that parameter optimization increases recall rather than precision, which is >0.95 in most simulations and SV types (Additional file 1: Figure S3). We also found that using a single set of (global) parameters optimized for all SV types in a given sample yields an accuracy that is as high as using a set of parameters specifically for each SV type (Additional file 1: Figure S4). This validates our approach of running SV calling once (with a single set of parameters) for each sample. Taken together, our results suggest that parameter optimization yields maximum performance by improving the recall of SVs as compared to default parameters.

**Fig. 3:**
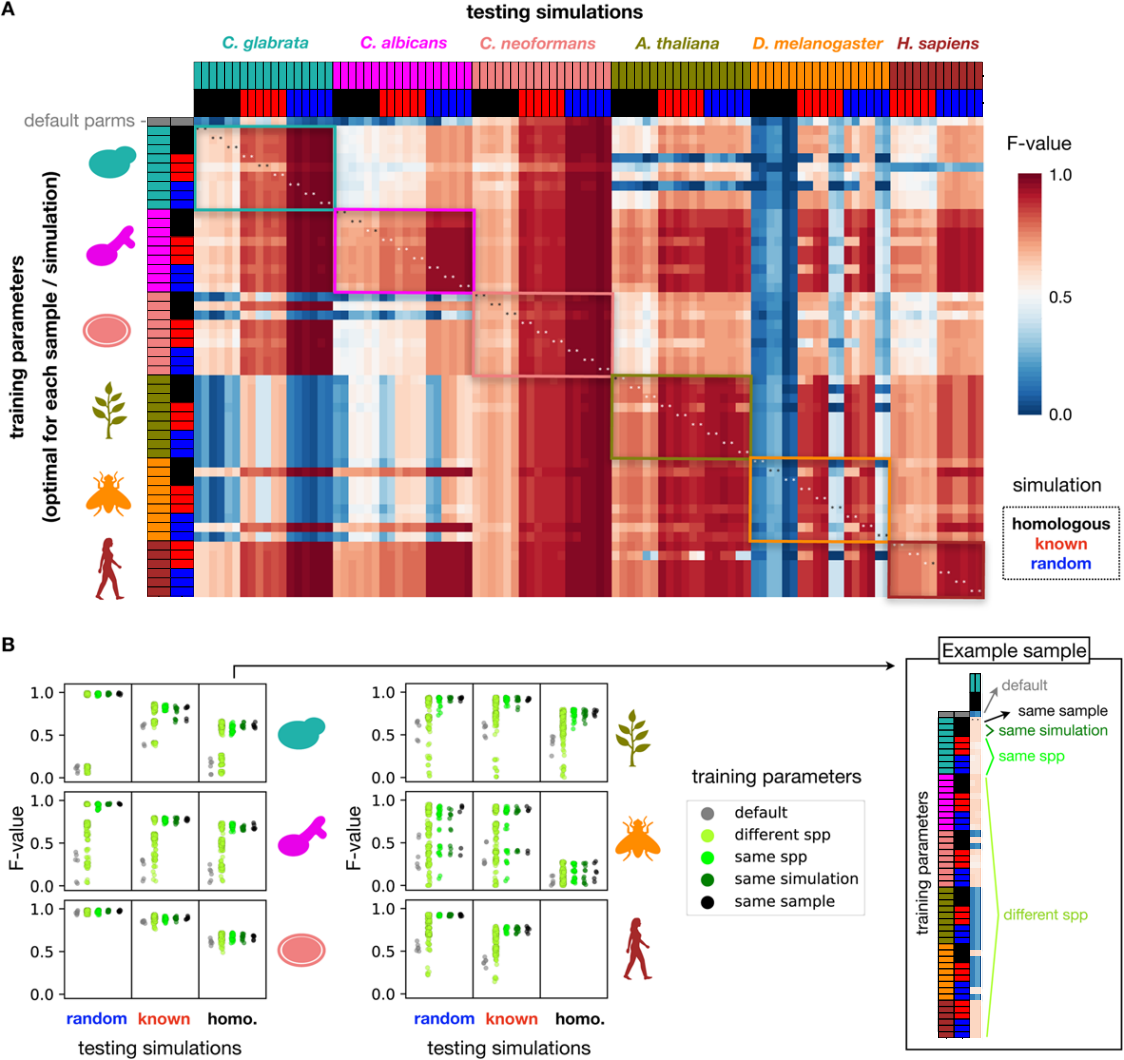
There is no universal recipe for SV calling across all species. **A** In order to assess whether perSVade’s parameter optimization is necessary for a given combination of sample and simulation (mentioned in Fig. 2), we measured the SV calling accuracy of each optimized parameter set on the other combinations. Each row indicates a different “training” parameter set optimized for each sample and simulation type in all species. In addition, the first row refers to the default parameters. Each column represents a simulation from a given sample / simulation type to be “tested.” The heatmap shows the *F*-value of each parameter set on each tested simulation (hereafter referred to as “testing instance”). Note that the species are ordered alike in rows and columns. In addition, note that each sample (from a given species and simulation type) yielded one set of training parameters and two simulated genomes tested here, which explains why there are two columns for each row. The colored boxes indicate testing instances where the training and testing species are equal. The asterisks refer to instances where both the sample and type of simulation are equal in the training and testing (equivalent to the “optimized” parameters from Fig. 2). Note that Additional file 1: Figure S6 shows the corresponding precision and recall, from which the *F*-value is calculated. **B** We summarized the data shown in **A** to compare how similar types of training parameters performed on each species (in the rows) and type of simulations (in the columns). Each point corresponds to a testing instance, matching one cell from the heatmap in **A**. The “default” and “same sample” reflect testing instances where the training parameters were either un-optimized or optimized specifically for each sample, respectively. The “different spp” group includes instances where the training parameters were from different species. The “same spp” group shows testing instances with both training parameters and tested simulations from a different sample of the same species. The “same simulation” reflects instances with the same training and testing sample, but different simulation types. For clarity, the right box shows how the training parameters are grouped for a set of “homologous” simulations based on one example *C. glabrata* sample (which corresponds to the first two columns in **A**)

We next explored whether different runs of perSVade (i.e., in different species or simulation types) yield similar parameters, which may clarify how necessary this optimization is. We hypothesized that each sample and simulation type combination may require specific parameters that would not necessarily work for other samples. To test this, we first compared the chosen parameters across different runs, which appeared to be sample-specific (Additional file 1: Figure S5A). This suggests that there is not a universal recipe (i.e., filtering parameters) for SV calling with perSVade. However, another (null) hypothesis could be that different parameter sets have similar outcomes, without changing the SV calling accuracy. This question was highly important to us. If perSVade’s optimization converges to equivalent parameter sets in different samples we would not need the optimization on every sample (i.e., we could re-define one of these parameters as default). In order to sort this out, we evaluated how different parameter sets (either “default” ones or those that are defined as “optimum” for a given sample) work on simulated genomes related to other samples. The results of this analysis are shown in Fig. 3 and Additional file 1: Figure S6. As hypothesized, not all the parameter sets yield accurate results on all samples, with large differences between species (Fig. 3A). However, we found that parameters optimized for one sample are mostly accurate on samples of the same species, regardless of the simulation type (Fig. 3B). Of note, the parameters yielded by “random” simulations were accurate on “homologous” and “real” simulations (Fig. 3). This indicates that running perSVade on “random” simulations (the cheapest setting in terms of resources) yields accurate parameters for more realistic simulations and possibly real SVs. On another line, we found that the different parameters changed mostly the SV calling recall, and not the precision (Additional file 1: Figure S6).

To understand why certain parameter choices impact SV calling accuracy, we tested how changing each parameter in isolation (keeping all others with default values) affects accuracy in these different species and simulations (Additional file 1: Figure S5). We first used these data to assess whether the change of single parameters drives the optimization process. We measured, for each parameter and sample, the ratio between the single-parameter-change *F*-value (where only one parameter has the optimal value) and the maximum *F*-value (obtained with the set of parameters where all parameters are optimized). We find that 78.05% of these parameter-sample instances have an *F*-value ratio below 0.75 (Additional file 1: Figure S5B), suggesting that the optimal accuracy is mostly reached by a complex interplay between different (at least 2) parameters, rather than being driven by a single-parameter change.

This analysis also serves to evaluate the impact of different parameters on SV calling accuracy. For example, we find that the set of vcf “FILTER” tags defining accepted breakpoints (*wrong_FILTERtags* parameter) drastically affects the accuracy in *C. glabrata*, in a way that requiring de novo assembly support for breakpoints (default behavior) is too conservative. A similar (but smaller) effect is observed in *C. albicans*, but not in the other species, which could be due to unique genomic features and/or technical properties in the *Candida* samples driving worse assembly performance. In addition, the coverage thresholds that define tandem duplications and deletions (*min_rel_coverage_dup* and *max_rel_coverage_del*, respectively) determine accuracy in a way that is dependent on ploidy, likely because diploid species (*C. albicans*, *D. melanogaster*, and *H. sapiens*) require a less conservative threshold to accept heterozygous variants. Importantly, these three parameters (*wrong_FILTERtags*, *min_rel_coverage_dup* and *max_rel_coverage_del*) explain why default parameters are suboptimal in most cases, as the default values can be too conservative in different species. On another line, the minimum number of supporting reads per variant (*min_Nfragments*) changes accuracy, with sample-specific effects (see *D. melanogaster* and *A. thaliana*), which we attribute to varying coverage, read lengths, or sequencing quality. Finally, filtering out variants that overlap any repetitive elements (*filter_overlappingRepeats*) generally reduces accuracy for realistic simulations (“homologous” and “known”), likely due to the fact that real variants could appear around such repeats. Conversely, there are other parameters that have minimal effects on accuracy (*dif_between_insert_and_del*, *filter_noReadPairs*, *max_to_be_considered_small_event*, *maximum_length_inexactHomology*, *maximum_microhomology*, *maximum_strand_bias*, *min_QUAL*, *min_af*, *min_length_inversions*, *range_filt_DEL_breakpoints*) (Additional file 1: Figure S5B). However, these parameters can have an impact in some samples and, since perSVade only considers parameter values that can change the filtering in each sample (see “Methods”), we consider that they should not be removed from the “optimize_parameters” module.

Our analysis also showed that the need for parameter optimization is different for each species. An illustrative example is the dramatic difference between *C. neoformans* and *C. glabrata* (Fig. 3A), which provides further insights on the role of various parameters. We found that the parameter choice is irrelevant in *C. neoformans*, while *C. glabrata* samples required specific optimization (Fig. 3A). We consider that this is unlikely driven by intrinsic genomic differences between the two species, as both have small (<20Mb) haploid genomes with low content of simple repeats (0.98% in *C. glabrata* and 0.80% in *C. neoformans*) or low-complexity regions (0.16% in *C. glabrata* and 0.21% in *C. neoformans*). We hypothesized that *C. glabrata* samples have an excessively high coverage (>300×, while *C. neoformans* samples have a 30×–40× coverage (Additional file 1: Table S1)) which may constrain SV calling accuracy and require optimized parameters. To test this, we measured the accuracy of different parameter sets on the *C. glabrata* simulations with randomly downsampled coverages. As hypothesized, we find that most parameters are accurate on the *C. glabrata* with 30× coverage, while simulations with lower (10×) and higher (100×–500×) coverage require specific parameters (Additional file 1: Figure S7). These results suggest that 30×–40× could be the optimal coverage for perSVade, which is reasonable given that *gridss* was developed for human datasets with similar coverage. However, there are still differences between the *C. neoformans* and the downsampled (30×) *C. glabrata* samples. For example, there are two parameter sets optimized for the high-coverage *C. glabrata* samples (both requiring at least 30 supporting reads per SV) which are accurate on all *C. neoformans* simulations (Fig. 3A), but not on the *C. glabrata* 30× (Additional file 1: Figure S7). This suggests that there are different genomic features between these species (i.e., content of simple repeats) constraining the accuracy. These findings indicate that both technical variation (i.e., changes in coverage) and different genomic features underlie the observed differences in SV calling accuracy between species. Importantly, this also illustrates that perSVade can adapt to each sample and yield optimal results.

In summary, our results suggest that parameter optimization is necessary for maximum performance in each species and dataset and that there is a complex interplay between parameters.

### PerSVade’s parameter optimization improves the calling accuracy in datasets with defined sets of real SVs

The performance of SV calling on simulations may not be equivalent on real data, as SVs often appear around repetitive or low-complexity regions which hamper their detection [24, 26–28]. It is thus possible that we overestimated the real accuracy in our simulations. We partially addressed this with our analysis based on “realistic” simulations (“known” and “homologous”), where the inferred accuracy was lower (Fig. 2) and potentially closer to the real one. To further validate the usage of perSVade for real SV calling, we tested it on datasets with known SVs, which were available for the human samples tested above (i.e., Fig. 3). We ran perSVade (using different simulation types) on the same three datasets, which had previously defined deletions and inversions (see “Methods” for details).

**Fig. 4:**
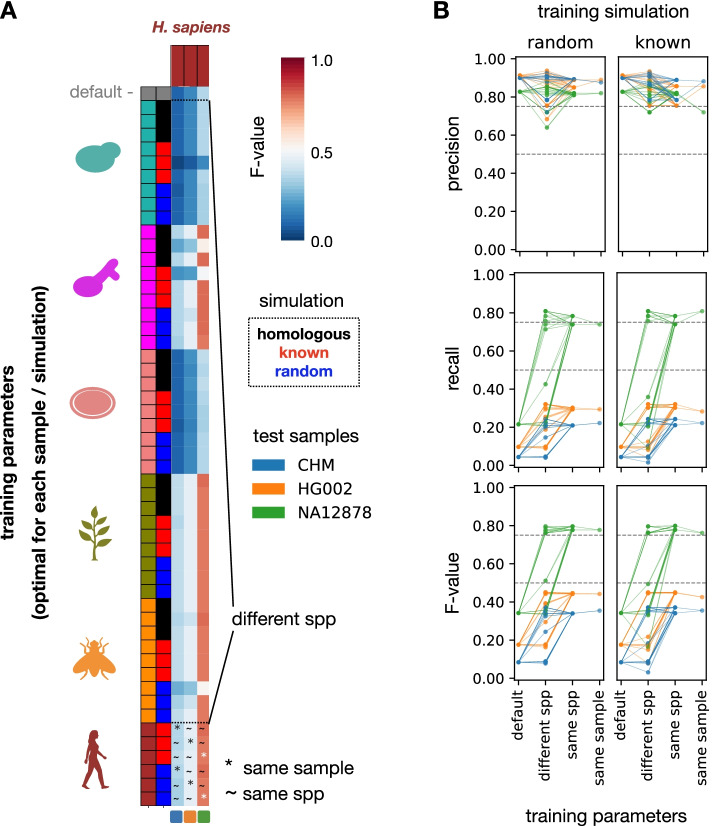
PerSVade’s parameter optimization improves the SV calling accuracy on datasets with known real SVs. **A** To test perSVade’s performance on real SVs, we measured how the parameters optimized for several simulations in different species (see Fig. 3) work on three human samples (CHM, HG002, and NA12878) with defined sets of real SVs. Each row indicates one of these different “training” parameters optimized for each sample and simulation type. In addition, the first row refers to the default parameters. Each column represents a sample with defined real SVs to be “tested.” The heatmap shows the *F*-value of each parameter set on each tested real sample (hereafter referred to as “testing instance”). In addition, we divide the testing instances into different groups (“default,” “different spp,” “same spp,” and “same sample”), which are relevant to understand the **B** panel. The “different spp” group refers to instances where the training and testing species were different. The “~” (same spp) refers to instances where the training and testing samples were different, but from the same species. Finally, the “*” (same sample) refers to instances where the training and testing samples were the same. **B** We summarized the data shown in **A** to compare how similar types of training parameters performed on each testing sample (each represented by a different color). Each row corresponds to a different accuracy measure. Each point corresponds to a testing instance (matching one cell from the heatmap in **A** in the bottom “*F*-value” plots). The “default” and “same sample” reflect testing instances where the training parameters were either un-optimized or optimized specifically for each sample, respectively. The “different spp” group includes instances where the training parameters were from a different, non-human, species. The “same spp” group shows testing instances with both training parameters and tested simulations from different samples of the same species. In addition, each column represents testing instances where the training parameters were based on “random” or “known” simulations, respectively. Note that the different groups of “training parameters” are equivalent to those shown in **A**

We used these data to assess the accuracy of perSVade on real datasets, using different sets of parameters (optimal for each simulation and sample from the six species tested above, shown in Fig. 3). As expected, we found a lower *F*-value on real datasets (Fig. 4) as compared to the simulated genomes (Figs. 2 and 3), with high precision and lower recall (Fig. 4B). In addition, parameter optimization improved the *F*-value modulating both precision and recall (Fig. 4B). However, the other results described in the simulations’ analysis (related to the performance of the pipeline and the universality of the parameters) are qualitatively equivalent in these real datasets (Fig. 4). Taken together, our analysis indicates that perSVade improves SV calling in real datasets (similarly to simulated genomes).

## Discussion

Despite large variation of genomic features across taxa, SV detection approaches in non-model organisms tend to rely on tools and parameters developed for other species (generally human). We hypothesized that this “one size fits all” approach is suboptimal, and likely biased towards certain species. To test this idea and overcome the problem, we developed perSVade, a flexible pipeline that automatizes the calling and filtering of structural variants (SV) across eukaryotes. PerSVade is a modular method to automatically adapt a state-of-the-art SV calling pipeline to any sample/species of interest. PerSVade uses simulations to choose the optimal filters for each sample and report the calling accuracy, which can inform about the reliability of the results. This will allow users to be aware of the accuracy in their datasets (i.e., perSVade may be inaccurate in some datasets due to low coverage, short read lengths, or excessive repeats in the genome) and make informed choices.

We validated the broad usability of perSVade by testing it on simulations and real datasets for a wide range of eukaryotes (with genomes of 12–3000 Mb and datasets including 10^7^–10^9^ reads). We found that there is a significant computational burden related to parameter optimization, which may hinder its usage on large genomes. This means that perSVade may be particularly cost-effective for small genomes (i.e., <200 Mb). However, the chosen settings will likely depend on the available resources, and some users may consider that the resources spent (see Additional file 1: Figure S2) are worth it given that parameter optimization yields improved accuracy (see below).

This testing also revealed that, as we hypothesized, parameter optimization improves the calling accuracy on both simulations and datasets with real, previously defined SVs. We found that the optimization mostly improves the recall rather than precision (which is generally high regardless of the used parameters). However, there are some exceptions (mostly in the testing on real SVs), suggesting that optimization can be necessary for reaching both high recall and precision in some samples. In addition, perSVade’s optimization yielded unique parameter sets for each sample, which were often inaccurate on other datasets. This means that there is no universal set of parameters that work well for all samples, which justifies the need for parameter optimization and a tool like perSVade to automate such a task. Conversely, we found some trends that can be useful to skip parameter optimization in some cases. For instance, parameter sets were often accurate across datasets of the same species (which could be due to differences in coverage and/or intrinsic genomic features). In addition, parameters resulting from “random” simulations performed well in more realistic (“known” and “homologous”) simulations as well as in real SV datasets of the same species, indicating that they can be used for maximum performance. Based on these findings, we propose the following recommendations for a cost-effective usage of perSVade:

- For SV calling on many datasets of one species with similar properties (similar coverage, read length, and insert size), run perSVade using “random” simulations on one sample, and use the optimized parameters for the other samples (skipping optimization). The reported calling accuracy may be overestimated since the simulations are not realistic, but the chosen parameters are expected to be optimal. This strategy may be particularly suited to large genomes, where users would avoid the computational burden of optimizing parameters for each sample.
- For approximating the real SV calling accuracy, run perSVade on realistic simulations (“homologous” or “known”), which may report an accuracy that is closer to the real one.
- For SV calling on large datasets, consider the following options to speed up the process. First, rationally design the parameters (based on parameters optimized for similar samples (see first point) and/or the benchmarking shown in this work) instead of inferring them with the “optimize_parameters” module for every new sample. Second, skip marking duplicates in read alignment, which can be very costly. Third, limit the simulations to a subset of chromosomes in the “optimize_parameters” module. Fourth, randomly downsample your reads (i.e., to 30×), which may improve both performance and accuracy (see Additional file 1: Figure S7).

We note that perSVade is not a fundamentally new algorithm for SV detection but rather a pipeline implementing existing algorithms. This is why we did not compare it with other such methods (like *manta* [20] or *delly* [29]). The novelty of our pipeline lies in the automatic parameter selection feature, which is unique (to the best of our knowledge) for short read-based SV calling. We thus centered our testing on the accuracy of different parameters on SV calling. In fact, some recent approaches specifically developed for human genomes [22, 30] may outcompete perSVade in human samples. However, such methods rely on previously defined sets of known SVs, which are not available in most taxa. We thus consider that our pipeline will be mostly useful in species without such specific methods available. For example, perSVade was used in a recent study to find SVs associated with antifungal drug resistance in the non-model yeast *Candida glabrata* [9], which successfully validated all (8/8) the predicted variants using PCR.

An open question is whether a similar parameter optimization strategy can be applied to SV calling pipelines based on other algorithms. Several studies have shown that the filtering strategies (considering parameters like read coverage, variant quality and vcf “FILTER” tags) largely affect the calling accuracy in various algorithms [23, 24]. This suggests that most SV callers could be boosted with a parameter optimization strategy such as the one described here for *gridss* and *clove*. However, due to high heterogeneity in SV callers, each algorithm may require a custom pipeline to deal with caller-specific parameters, outputs, and SV types.

Finally, perSVade also includes modules for CNV identification and SNP/INDEL calling, as a way to automate the finding of other broadly used genomic variants. In addition, it includes variant annotation features to ease the functional interpretation of these variants for downstream analyses. In summary, perSVade is a Swiss-knife-like framework to identify many types of variants with a few bash commands. We consider that this tool will be useful to understand the role SVs in different phenotypes and organisms, particularly those with no specific recommendations.

## Conclusions

- perSVade can identify SVs from short reads with sample-optimized parameters using a few bash commands.
- perSVade can predict the SV calling accuracy on simulated genomes, which informs about the reliability of the calling process in an automatic manner.
- perSVade’s parameter optimization improves the SV calling accuracy on simulated variants for five eukaryotic organisms, and on a reference dataset of validated human variants.
- We found no universal set of “optimal” parameters, which underscores the need for species-specific parameter optimization for SV calling.

## Methods

### PerSVade pipeline

PerSVade has several modules that can be executed independently (each with a single command) and/or combined to obtain different types of variant calls and functional annotations. The following sections describe how each of these modules work, and Fig. 1 shows how they can be combined.

#### Module “trim_reads_and_QC”

This module runs *trimmomatic* [31] (v0.38) with default parameters for the input reads followed by *fastqc* [32] (v0.11.9) on the trimmed reads. These trimmed reads may be used for downstream analysis after checking that they are reliable according to the output of *fastqc*.

#### Module “align_reads”

This module runs *bwa mem* [33] (v0.7.17) to align the short reads, generating a sorted .bam file (using *samtools* [34] (v1.9) with marked duplicates (through *GATK MarkDuplicatesSpark* [35] (v4.1.2.0)), that is the core input of several downstream modules (“call_SVs,” “optimize_parameters,” “call_CNVs,” “call_small_variants,” and “get_cov_genes”). If --*skip_marking_duplicates* is specified, this module skips the marking of duplicate reads (default behavior), which may be useful to speed up the process in large datasets.

#### Module “call_SVs”

This module uses *gridss* [21] to infer a list of breakpoints (two regions of the genome—two breakends—that are joined in the sample of interest and not in the reference genome) from discordant read pairs, split reads, and de novo assembly signatures. The breakpoints are summarized into SVs with *clove* [25] (v 0.7). Importantly, this module (and others) runs *clove* without the default coverage filter to classify deletion-like (DEL-like) and tandem duplication-like (TAN-like) breakpoints into actual deletions and tandem duplications. Instead, perSVade “call_SVs” calculates the relative coverage of the regions spanned by such breakpoints (using *mosdepth* [36]). This information is used to define the final set of deletions (DEL-like breakpoints with a coverage below a “max_rel_coverage_to_consider_del” threshold) and tandem duplications (TAN-like breakpoints with a coverage above a “min_rel_coverage_to_consider_dup” threshold). This setting allows a separate thresholding for the classification of DEL and TAN-like breakpoints, which is a novel feature of perSVade as compared to the current implementation of *clove*. Note that this module requires as an input a set of parameters to filter the *gridss* and *clove* outputs. These parameters may be inferred using the module “optimize_parameters” (described below) or rationally designed based on the benchmarking shown here (which can be useful to speed up the process). In addition, the user can use a set of default parameters, described in the section “Filters used by perSVade” below. Note that these default parameters are inspired by previous filtering strategies from [21, 37, 38].

The final output of this module is a set of files with the called variants (one file for each variant type), which belong to these types:

- Simple SVs: deletions, inversions, and tandem duplications (duplication of a region which gets inserted next to the affected region). This module outputs one .tab file for each of these SV types.
- Translocations: whole-arm balanced translocations between two chromosomes, which can be inverted or not. There is one .tab file for translocations.
- Insertions: a region of the genome is copied or cut and inserted into another region. Note that these are not de novo insertions (i.e., of DNA not present in the reference), which are actually not called in this module. There is one .tab file for insertions.
- Unclassified SVs: One .tab file reports all the variants that are called by *clove* and cannot be assigned to any of the above SV types. These include *clove*’s unclassified breakpoints (which could be part of unresolved/unknown complex variants) and complex inverted SVs (which are non-standard SVs). These types of SVs are not included in the simulations performed by “optimized parameters” (see below), so that their accuracy is unknown. This is why we group them together into a single file.

These separate files have a tabular format, where each variant is represented in a single line. In addition, the module “integrate_SV_CNV_calls” (see below) generates a single .vcf file with all the variants together, represented in a way that is focused on how each SV affects particular regions of the genome (useful for functional annotation). PerSVade’s github wiki [39] includes further information on the output formats.

On another line, note that *gridss* does report de novo insertions, but the usage of short reads limits the calling to small events, which may miss many real de novo insertions. This is why we decided to not consider such variants as a trustful output in this module or the “optimize_parameters.” However, “call_SVs” saves the raw *gridss* output, and the unfiltered small de novo insertions can be obtained (although these should be treated with caution). In addition, note that these *de novo* insertions are different from non-template insertions happening around the breakends of actual SVs. Non-template insertions are likely the product of DNA repair after the rearrangement, and they are considered in the “integrate_SV_CNV_calls” (see below).

#### Module “optimize_parameters”

To find optimal parameters for running “call_SVs” in a given input dataset, this module generates two template (haploid) simulated genomes (the number can be customized with *--nsimulations*) with up to 50 SVs of each of five types (insertions, translocations, deletions, inversion, and tandem duplications) with *RSVsim* [40] (v1.28) and custom python (v3.6) scripts (which use *biopython* [41] (v1.73)). By default, this template genome contains all chromosomes in the reference, but this can be customized with the *--simulation_chromosomes* argument to only simulate a subset of chromosomes and speed up the process. For each template genome, the module simulates reads with *wgsim* [42] (v1.0) and *seqtk* [43] (v1.3) with a read length, insert size, and coverage matching that of the input dataset. Note that the read simulation is performed according to a user-defined zygosity and ploidy (through the mandatory argument “*--simulation_ploidies*”) to resemble various organisms. For example, if “*--simulation_ploidies diploid_hetero*” is specified, this module simulates reads with heterozygous SVs by merging reads from both the reference genome and the simulated genome with SVs in a 1:1 manner. Importantly, *--simulation_ploidies* can have multiple values, so that for each template simulated genome and simulation ploidy this module generates unique simulated reads with the specified ploidy and zygosity. For example, if “--*nsimulations 2 --simulation_ploidies diploid_hetero*,*diploid_homo*” is set, this module generates four simulated reads. First it generates two template genomes, and for each of them it simulates reads with either heterozygous or homozygous SVs. Note that “*--simulation_ploidies*” can include any combination of “haploid,” “diploid_homo,” “diploid_hetero,” and “ref:<nref>_var:<nvar>” (where <nref> / <nvar> are the number of reference / alternative chromosomal copies, respectively). For example, setting “*--simulation_ploidies* ref:3_var:1” simulates reads assuming a tetraploid genome, where three chromosomes are like the reference and one has the SVs. This flexibility in setting ploidies / zygosity allows adapting this module to polyploid genomes or complex samples (i.e., pools of different samples of a population).

For each set of simulated reads (from one template genome with a specific ploidy and zygosity), perSVade “optimize_parameters” then tries several combinations (>278,000,000,000 by default, although this can be user-defined with the argument *--range_filtering_benchmark*) of parameters to run *gridss* and *clove* and filter their outputs. The detailed explanation about the used filters can be found in the section “Filters used by perSVade” below**.** To reduce the number of parameter combinations to be optimized, the pipeline discards parameter values that do not change breakpoint filtering as compared to an unconservative set of parameters. This means that the set of parameters to be optimized are limited to those that can be relevant, and these could be different in any run. One of these possible filters includes removing SVs that overlap repetitive elements, which may be inferred with the module “infer_repeats” (see below). This module selects the combination of filters that yield the highest *F*-value (the harmonic mean between precision and recall) for each SV type in each template simulated genome and ploidy/zygosity (see the section “Comparing sets of SVs to calculate precision and recall” below for more information on how accuracy is calculated). These filters are optimized for each simulation, and thus may not be accurate on independent sets of SVs (due to overfitting). In order to reduce this effect, perSVade “optimize_parameters” selects a final set of “best parameters” that work well for all simulations, ploidies/zygosities, and SV types. This set of best parameters may be used in the “call_SVs” module. The accuracy (*F*-value, precision, recall) of these parameters on each simulation and SV type is reported in a tabular file, which serves to evaluate the expected calling accuracy. Note that we default the number of template simulated genomes to two in order to have a meaningful evaluation of overfitting (which likely requires more than one template genome). In addition, note that setting several simulation ploidies can be useful to select parameters that work well for different ploidies/zygosities.

All plots are generated using *python* (v3.6) and the libraries *seaborn* [44] (v0.9.0) and *matplotlib* [45] (v3.3.0). In addition, the *python* packages *scipy* [46] (v1.4.1), *scikit-learn* [47] (v0.21.3), *psutil* [48] (v5.7.2), and *pandas* [49] (v0.24.2) are used for scripting and various statistical calculations. On another line, *pigz* [50] (v2.4) and *gztool* [51] (v0.11.5) are used for fast compression steps. Finally, perSVade “optimize_parameters” uses *picard* [52] (v2.18.26) to construct a sequence dictionary for the provided reference genome.

By default, the simulated events are placed randomly across the genome. However, real SVs often appear around repetitive elements or regions of the genome with high similarity (e.g., transposable elements insertions) [24, 26–28]. This means that random simulations may not be realistic, potentially leading to overestimated calling accuracy and a parameter selection inaccurate for real SVs [24]. To circumvent this, perSVade “optimize_parameters” can generate more realistic simulations occurring around some user-defined regions (i.e., with previously known SVs or homologous regions) provided with the --*regions_SVsimulations* argument. Importantly, perSVade provides an automatic way to infer such regions through the modules “find_knownSVs_regions” and “find_homologous_regions” (described below). Beyond setting custom regions, users may want to tune the number of simulated SVs (through the *--nvars* argument) to be realistic in the samples/species of interest. In addition, note that the variant size is proportional to genome length, which ensures that long genomes have larger sections under SV.

Finally, note that Additional file 1: Figure S1 includes a detailed graphical representation which can be useful to understand how this module works.

#### Module “find_knownSVs_regions”

This module finds regions with known SVs using a provided list of sequencing datasets (with the option *--close_shortReads_table*) from species close to the reference genome. These datasets are processed with perSVade’s modules “trim_reads_and_QC,” “align_reads,” and “call_SVs” (using default parameters) to find SVs. This module then outputs a .bedpe file with the ±100bp regions around the breakends from these SVs. This .bedpe file can be input to the module “optimize_parameters” through the *--regions_SVsimulations* argument in order to perform “known” realistic simulations.

#### Module “find_homologous_regions”

This module infers homologous regions by defining genomic windows (from the reference genome) of 500 bp as a query for a *blastn* [53] (v2.10.0+) against the same reference genome. Hits with an e-value <10^−5^ that cover >50% of the query regions are defined as pairs of homologous regions, which are written as a .bedpe file. This .bedpe file can be input to the module “optimize_parameters” through the *--regions_SVsimulations* argument in order to perform “homologous” realistic simulations.

#### Module “call_CNVs”

Copy number variants (CNVs) are a type of SVs in which the genomic content varies (deletions or duplications). The “call_SVs” module (see previous section) identifies some CNVs (insertions, tandem duplications, deletions, and complex inverted SVs) but it can miss others (i.e., whole-chromosome duplications or regions with unknown types of rearrangements yielding CNVs [8, 54]). PerSVade uses this “call_CNVs” module to call CNVs from read-depth alterations. For example, regions with 0× or 2× read depth as compared to the mean of the genome can be called deletions or duplications, respectively. A straightforward implementation of this concept to find CNVs is challenging because many genomic features drive variability in read depth independently of CNV [55, 56]. In order to solve this, perSVade “call_CNVs” calculates the relative coverage for windows of the genome (using *bedtools* [57] (v2.29.0) and *mosdepth* [36] (v0.2.6)) and corrects the effect of the GC content, mappability (calculated with *genmap* [58] (v1.3.0)), and distance to the telomere (using *cylowess* [59] for nonparametric regression as in [56]). Note that *cylowess* uses the library *cython* [60] (v0.29.21). This corrected coverage is used by *CONY* [61] (v1.0), *AneuFinder* [62] (v1.18.0), and/or *HMMcopy* [63] (v1.32.0) to call CNVs across the genome. Note that we modified the R code of *CONY* to be compatible with the input corrected coverage. The corrected code (used in the pipeline) is available in “scripts/CONY_package_debugged.R” from [39]. PerSVade “call_CNVs” generates consensus CNV calls from the (up to) three programs taking always the most conservative copy number for each bin of the genome. For example, if the used programs disagree on the copy number of a region the closest to 1 will be taken as the best estimate. Note that the parameters obtained in the module “optimize_parameters” cannot be used for this module, since the SV and CNV calling methods are fundamentally different.

#### Module “integrate_SV_CNV_calls”

This module generates a vcf file showing how SVs (called by the modules “call_SVs” and/or “call_CNVs”) alter specific genomic regions. We designed this vcf to be compatible with the Ensembl Variant Effect Predictor [64] (VEP) tool for functional annotation, which can interpret tandem duplication (TDUP) duplication (DUP), deletion (DEL), and breakend-like (BND) events. This requires the decomposition of each variant into such TDUP, DUP, DEL, and BND events (one event in each row of the vcf). For example, each inversion is decomposed into two BND events (two rows in the vcf), one for each end of the inversion. The rationale behind this is that, in terms of functional annotation for inversions, we are interested in genomic features that are around the ends of the inversion, where the rearrangement happens. Each SV can thus be split across multiple rows when it affects more than one region of the genome. All rows related to the same SV are identified by the field variantID in INFO. On top of this, each row has a unique identifier indicated by the field ID. Some SVs generate non-template inserted sequences around the breakends (likely the product of DNA repair after a rearrangement), and each of these is represented in a single row. Note that each of the rows may indicate a region under CNV (with the SVTYPE in INFO as DEL, DUP, or TDUP), a region with some breakend (with the SVTYPE in INFO as BND) or a region with a non-template insertion (with the SVTYPE in INFO as insertionBND) around the breakend. Such non-template insertions are included here because they may modulate the impact of SVs on genomic features, and thus they are relevant for functional annotation. Note that this module also removes redundant calls between the CNVs identified with “call_SVs” (tandem duplications, deletions and insertions) and those derived from “call_CNVs.” To remove redundancy, this module skips any CNV called by “call_CNVs” that overlaps reciprocally (by at least an 80% of the region) a CNV called by “call_SVs” using *bedmap* from the *bedops* tool [65] (v2.4.39). See the FAQ “What is in SV_and_CNV_variant_calling.vcf?” from [39] for more information about the format of this .vcf file.

#### Module “annotate_SVs”

This module runs the Ensembl Variant Effect Predictor [64] (v100.2) on the vcf output of the module “integrate_SV_CNV_calls” to get the functional annotation of each SV. This requires a .gff file from the user.

#### Module “call_small_variants”

This module performs small variant (SNPs and small IN/DELs) calling with either *freebayes* [66] (v1.3.1), *GATK HaplotypeCaller* [67] (v4.1.2.0), and/or *bcftools call* [68] (v1.9) and integrates the results into .tab and .vcf files. The section “Calling of small variants” below provides further information on how this calling is performed.

#### Module “annotate_small_vars”

This module runs the Ensembl Variant Effect Predictor [64] (v100.2) on the vcf output of the module “call_small_variants” to obtain the functional annotation of each variant. This requires a .gff file from the user.

#### Module “get_cov_genes”

This module runs *mosdepth* [36] (v0.2.6) to obtain the coverage for each gene, which requires a .gff file from the user.

#### Module “infer_repeats”

This module annotates repetitive elements in a genome, which can be used for the modules “call_SVs,” “find_knownSVs_regions,” “integrate_SV_CNV_calls,” “optimize_parameters,” and “call_small_variants.” These repeats are inferred with RepeatModeler [69] (v2.0.1) and RepeatMasker [70] (v4.0.9). The user can input these repeats to several modules (with --repeats_file), which will have the following effects:

- If repeats are provided, “optimize_parameters” will assess whether removing SV calls overlapping repeats increases the overall accuracy. If so, the resulting optimized parameters will include a “filter_overlappingRepeats : True.” If you use these optimized parameters in “call_SVs,” any breakpoint overlapping repeats will be removed.
- If repeats are provided, “call_SVs” may filter out SVs that overlap repeats if the SV filtering parameters include a “filter_overlappingRepeats : True.”
- If repeats are provided, “find_known_SVs” will pass them to the “call_SVs” module.
- If repeats are provided, “integrate_SV_CNV_calls” will add a field in the INFO which indicates whether the SVs overlap repeats.
- If repeats are provided, “call_small_variants” will add a field in the tabular variant calling file which indicates whether the SVs overlap repeats.

Alternatively, the user can specify “--repeats_file skip” to avoid the consideration of repeats in all these modules.

### Testing SV calling with perSVade on simulated structural variants

To test perSVade’s performance on different species, we ran it on paired-end WGS datasets for six eukaryotes (*Candida glabrata*, *Candida albicans*, *Cryptococcus neoformans*, *Arabidopsis thaliana*, *Drosophila melanogaster*, and *Homo sapiens*). To obtain a high number of SVs, we gathered three samples for each species with enough genetic divergence to the reference genome. For this, we first used an automatic pipeline to find these samples running the custom script “scripts/perSVade.py” from [39] with the options *--close_shortReads_table auto --n_close_samples 3 --nruns_per_sample 1 --target_taxID <species_taxID>*. This used *entrez-direct* [71] (v13.3), *SRA Tools* [72] (v2.10.9), and *ete3* [73] (v3.1.2) to query the SRA database [74] and find three WGS datasets of close taxIDs (to each *<species_taxID>* according to the NCBI taxonomy species tree [75]) with a coverage >30× and >40% of mapped reads to the reference genome. We could find three such datasets for *C. albicans*, *C. neoformans*, *A. thaliana*, and *D. melanogaster*, which included samples from the same species or genera as the target species, with >65% of the reads mapping to the reference genome. We randomly downsampled the *A. thaliana* and *D. melanogaster* runs to 30× coverage (using *samtools* [34] (v1.9)) for faster computation (using the option *--max_coverage_sra_reads 30*). For *C. glabrata*, we used datasets generated in our lab from three divergent strains (BG2, CST34, and M12, from [9]). All these datasets are listed in Additional file 1: Table S1. Finally, we tested perSVade on three *H. sapiens* datasets previously used for benchmarking SV callers [23, 24]. These included NA12878 (a Genome in a Bottle (GIAB) cell line related to the Ceph family [76, 77]), HG002 (another GIAB project with reads available at [78]), and CHM1/CHM13 (two haploid cell lines sequenced independently [79], for which we merged the raw reads to generate synthetic diploid data). Note that we chose testing datasets with various read lengths and coverages (see Additional file 1: Table S1) to evaluate how perSVade works on realistic diverse scenarios. The reference genomes were taken from the Candida Genome Database [80] (version s02-m07-r35 for *C. glabrata* and “haplotype A” from version A22-s07-m01-r110 for *C. albicans*), GenBank [81] (accession GCA_000149245.3 for *C. neoformans*, GCA_000001735.2 for *A. thaliana* and GCA_000001215.4 for *D. melanogaster*), and UCSC [82] (the latest version of genome hg38 at 06/04/2021 for *H. sapiens*, keeping only chromosomes 1-22, X,Y and the mitochondrial DNA). In addition, we performed quality control of the reads with fastqc [32] (v0.11.9) and trimming with *trimmomatic* [31] (v0.38).

We ran the SV calling pipeline of perSVade (using the modules “align_reads,” “call_SVs,” and “integrate_SV_CNV_calls”) on all these datasets using either “default” or optimized parameters (based on “random,” “known,” or “homologous” simulations using the modules “optimize_parameters,” “find_homologous_regions,” and “find_knownSVs_regions”). Note that the default parameters were designed as a baseline to understand the need for parameter optimization. We thus pre-defined these parameters based on standard author recommendations (from previous filtering strategies designed by the *gridss* authors [21, 37, 38]). By comparing the results of such parameters (designed based on previous usage) and the optimized ones, we could assess the gain in SV calling accuracy associated with parameter optimization. In addition, note that we used the module “infer_repeats” to find repetitive elements in each genome. These were provided to “optimize_parameters” to assess whether filtering out repeats improved SV calling accuracy. In addition, we simulated diploid heterozygous SVs for the diploid genomes (*C. albicans*, *A. thaliana*, *D. melanogaster*, and *H. sapiens*) and haploid SVs for the haploid genomes (*C. glabrata*, *C. neoformans*). Note that we decided to only simulate heterozygous variants in the diploid genomes to create the most challenging scenario for SV calling (since homozygous variants are expected to be easier to find due to higher coverage), as previously done [24]. In addition, the output of the “infer_repeats” module was used to calculate the fraction of the genome with simple repeats or low-complexity regions of *C. glabrata* and *C. neoformans.* We used computational nodes in an LSF cluster (https://www.ibm.com/support/pages/what-lsf-cluster) with 16 cores and either 32 Gb (for *C. glabrata*, *C. albicans*, *C. neoformans*), 64 Gb (for *A. thaliana* and *D. melanogaster*), and 96 Gb (for *H. sapiens*) of RAM for the testing. We first ran the read alignment step (module “align_reads”) for all samples, and then used the resulting .bam files as inputs for the other perSVade modules. We calculated the resource consumption (running time and maximum RAM used) for each of these perSVade runs, thus ignoring the resources related to read alignment. Of note, perSVade was run with different parameters for the human datasets to avoid excessive resource consumption and match our computational infrastructure. First, we skipped the marking of duplicate reads on the .bam files (default behavior) with perSVade’s *--skip_marking_duplicates* option on the module “align_reads.” Second, we ran the simulations on a subset of the genome (only chromosomes 2, 7, 9, X, Y and mitochondrial), by using the *--simulation_chromosomes* argument of the “optimize_parameters” module. Third, we skipped the “homologous” simulations in human samples because we could not finish the inference of pairs of homologous regions (see previous section) due to excessive memory consumption. By running this inference on a few chromosomes, we realized that there are millions of such regions, generating excessively large files. Note that this strategy may be used in general to speed up parameter optimization.

Finally, we tested the accuracy of all the optimized parameters (for each sample / simulation) on the other samples / simulations using the script “testing/get_accuracy_parameters_on_sorted_bam.py” from [39]. In addition, to test the impact of changing each parameter in isolation, we generated sets of parameters where only one parameter is changed to a non-default value. We then used this same script (“testing/get_accuracy_parameters_on_sorted_bam.py” from [39]) to measure the accuracy of each parameter set on each sample / simulation. On another line, to assess whether the high coverage of *C. glabrata* samples (>300×, see Additional file 1: Table S1) constrained SV calling, we measured the accuracy of each parameter set (optimized for each species / simulations) on the *C. glabrata* simulations with varying coverage. For each simulation (based on a sample and a type of simulation (homologous / known / uniform)), we subsampled randomly the reads to get a coverage of 10×, 30×, 50×, 100×, 200×, or 300× using *samtools* [34] and *mosdepth* [36] on the aligned simulated reads. We then used our custom script “testing/get_accuracy_parameters_on_sorted_bam.py” from [39] to test the SV calling accuracy on each downsampled simulation. The section “Comparing sets of SVs to calculate precision and recall” below provides further information on how accuracy is calculated.

### Testing perSVade on real SVs

To validate the usage of perSVade on real data, we focused on public datasets with available short reads and independently defined sets of known SVs. We could find such SVs in the human samples (also used in the testing mentioned above), for which SV callsets of deletions or inversions exist (as done in [24]). We defined as “true SVs” the deletions of NA12878 (defined in [77], available at [83]), the high-confidence deletions of HG002 (available at [84]) and the union of all deletions and inversions found in either CHM1 or CHM13 lines (defined by [79], available at [85]).

We then tested the accuracy of the “training” parameters optimized for each sample and simulation of the six eukaryotes mentioned above (in the section “Testing SV calling with perSVade on simulated structural variants”) on these human samples using our custom script “testing/get_accuracy_parameters_on_sorted_bam.py” from [39]. In addition, we removed SVs overlapping simple repeats or low-complexity regions (as inferred by the module “infer_repeats”) from this analysis. Note that each of these “true SV” datasets were defined on different reference genomes: the NA12878 and HG002 callsets were based on hg19 and the CHM1/CHM13 was relative to hg38. This means that we could not directly use the optimized training parameters from the human samples from the previous section, since they were all based on hg38. We thus ran perSVade’s SV calling and parameter optimization modules on NA12878 and HG002 using the hg19 reference, and used the resulting optimum parameters as “training” for these two samples. For this, we obtained the latest version of hg19 and hg38 genomes at 06/04/2021 from UCSC [82], keeping only chromosomes 1-22, X,Y, and the mitochondrial DNA.

### Filters used by perSVade

These are the filters used in the module “call_SVs,” whose values may vary across parameter optimization in perSVade (note that most of the *gridss* filters were inspired by the filtering strategy used to generate the somatic call set from [21, 37] and the original *gridss* paper [37, 38])):

- min_Nfragments: Minimum number of reads supporting a breakend in *gridss* to be accepted (default is 5).
- min_af: Minimum variant allele frequency of a breakend in *gridss* to be accepted (default is 0.25).
- min_af_EitherSmallOrLargeEvent: Minimum variant allele frequency (VAF) of a breakend in *gridss* to be accepted, regardless of how VAF is calculated (default is 0.25). Note that VAF is calculated differently depending on if the breakend spans a region longer than the insert size or not (see https://github.com/PapenfussLab/gridss/issues/234#issuecomment-521489484). We regularly (i.e., for the min_af filter) calculate a VAF assuming that the breakend is a small event (vaf_small) or a large event (vaf_large). If the length of the breakpoint is above a certain threshold, related to the insert size (“median insert size + median absolute deviation of the insert size”), perSVade sets VAF to be “vaf_large” and vice versa. Note that our distinction between small and large breakends could be error prone in some cases, and min_af_EitherSmallOrLargeEvent allows the filtering based on VAF independently of the size of the event. If min_af_EitherSmallOrLargeEvent is above 0, breakends that have both “vaf_small” and “vaf_large” below the set min_af_EitherSmallOrLargeEvent will be discarded.
- min_QUAL: Minimum quality (QUAL field of the vcf file) of a breakend in *gridss* to be accepted (default is 0).
- max_to_be_considered_small_event: Maximum length of a breakpoint in *gridss* to be considered a small event (default is 1000). Events shorter than this value are considered as “small events,” which are treated particularly by other filtering steps.
- min_length_inversions: Minimum length of inversion-like breakends in *gridss* to be accepted (default is 40).
- maximum_lenght_inexactHomology: Maximum length of the inexact homology region around a breakend in *gridss* to be accepted (default is 50). This filter is not applied to “small events,” as defined by “max_to_be_considered_small_event.”
- maximum_microhomology: Maximum length of the exact homology (microhomology) region around a breakend in *gridss* to be accepted (default is 50).
- maximum_strand_bias: Maximum strand bias of a breakend in *gridss* to be accepted (default is 0.99). This filter is only applied to “small events,” as defined by “max_to_be_considered_small_event.”
- filter_noReadPairs: Discards *gridss* breakends without discordant read pair support (default is false). This filter is not applied to “small events,” as defined by “max_to_be_considered_small_event.”
- filter_noSplitReads: Discards *gridss* breakends without split-read evidence (default is false). This filter is only applied to “small events,” as defined by “max_to_be_considered_small_event.”
- filter_overlappingRepeats: Discards *gridss* breakends overlapping repetitive elements (default is false). This will only have an effect if you provide a repeats file as inferred by the module “infer_repeats.”
- filter_polyGC: Discards *gridss* breakends with long inserted G or C sequences (>15bp) (default is true).
- wrong_FILTERtags: A set of values in the FILTER field of the *gridss* vcf which flag discarded breakends (default is [“NO_ASSEMBLY”]).
- range_filt_DEL_breakpoints: A range of lengths in which DEL-like breakends (as defined by *gridss*) are discarded if the breakend has a region with inexact homology above 5bp (default is [0, 1]). For example, if set to [500, 1000], DEL-like breakends whose length is between 500 and 1000bp with an inexact homology sequence >5 bp would be discarded.
- dif_between_insert_and_del: The margin given for comparing the length of the inserted sequence (len_seq) on a *gridss* DEL-like breakend and the length of the actual event (len_event) (default is 5). If len_seq > (len_event - dif_between_insert_and_del), the breakend is filtered out. This filter is only applied to “small events,” as defined by “max_to_be_considered_small_event.”
- max_rel_coverage_to_consider_del: The maximum relative coverage that a region spanning a DEL-like breakpoint (as defined by *clove*) can have to be classified as an actual deletion (default is 0.1). Note that the default is a conservative filter adapted to haploid genomes or homozygous variants.
- min_rel_coverage_to_consider_dup: The minimum relative coverage that a region spanning a TAN-like breakpoint (as defined by *clove*) can have to be classified as an actual tandem duplication (default is 1.8). Note that the default is a conservative filter adapted to haploid genomes or homozygous variants.

Note that all the breakpoints that have at least one breakend that does not pass the filters are discarded by perSVade.

### Calling of small variants

PerSVade’s small variant calling pipeline (module “call_small_variants”) uses three alternative methods (GATK Haplotype Caller (HC) [67] (v4.1.2), freebayes (FB) [66] (v1.3.1), and / or bcftools (BT) [68] (v1.9)) to call and filter single-nucleotide polymorphisms (SNP) and small insertions/deletions (IN/DEL) in haploid or diploid configuration (specified with the *-p* option). The input is the bam file generated by the “align_reads” module. This module defines as high-confidence (PASS) variants those that are in positions with a read depth above the value provided with *--min_coverage*, with extra filters for HC and FB. For HC, it keeps as PASS variants those where (1) there are <4 additional variants within 20 bases; (2) the mapping quality is >40; (3) the confidence based on depth is >2; (4) the phred-scaled *p*-value is <60; (5) the MQRankSum is >−12.5, and (6) the ReadPosRankSum is >−8. For FB, perSVade “call_small_variants” keeps as PASS variants those where (1) quality is > 1 or alternate allele observation count is > 10, (2) strand balance probability of the alternate is > 0, (3) number of observations in the reverse strand is > 0, and (4) number of reads placed to the right/left of the allele are > 1. Then, bcftools (v1.10) and custom python code are used to normalize and merge the variants called by each software into a consensus variant set, which includes only those variants called with high-confidence by *N* or more algorithms This results in one .vcf file with the high-confidence variants for each *N*. Note that this .vcf file only keeps variants for which the fraction of reads covering the alternative allele is above the value provided with *--min_AF* (which may be 0.9 for haploids or 0.25 for diploids). For diploid calls, it defines the genotype with the strongest support (the one called by most programs). In addition, the quality of each variant is calculated from the mean of the three algorithms. Beyond the filtered variant calls, this module writes a tabular file with all the raw variants with various metadata columns (i.e., the programs that called the variant), which can be used to apply a custom filtering of the variants.

### Comparing sets of SVs to calculate precision and recall

To measure accuracy in different sets of “called SVs” (in perSVade’s simulations and also the testing of the pipeline (related to Figs. 2, 3, and 4 and Additional file 1: Figures S1, S3, S4, S5, S6, S7)), we compared them against the corresponding sets of “known SVs” and calculated the following estimates:

$$\mathrm{Precision}=\mathrm{TP}/\left(\mathrm{TP}+\mathrm{FP}\right)$$

$$\mathrm{Recall}=\mathrm{TP}/\left(\mathrm{TP}+\mathrm{FN}\right)$$

$$F \text{-} \mathrm{value}=\left(2\times \mathrm{precision}\times \mathrm{recall}\right)/\left(\mathrm{precision}+\mathrm{recall}\right)$$

where true positives (TP) are those in the “called SVs” that match at least one variant from the “known SVs,” false positives (FP) are those in the “called SVs” that do not match any from the “known SVs,” and false negatives (FN) are those in the “known SVs” that are not matched by any variant from the “called SVs.” We define that two SVs are “matching” using a different approach for each type of SV:

- Inversions, tandem duplications, and deletions: both SVs are in the same chromosome, their altered regions are overlapping by 75% and their breakends are <50bp apart.
- Insertions: both SVs have the same origin and destination chromosomes and are both either cut-and-paste or copy-and-paste. In addition, the regions of the origin chromosome are overlapping by 75% and the breakends are <50bp apart. Finally, the starts of the destination chromosomes (insertion sites) in both SVs are <50bp apart.
- Translocations: both SVs have the same origin and destination chromosomes and are both either inverted or not. In addition, the breakpoint positions in both SVs are <50bp apart.

In addition, we calculated “integrated” precision and recall measures (related to Figs. 3 and 4 and Additional file 1: Figure S6) merging all the variants together into single sets of “called SVs” and “known SVs.” We used custom python (v3.6) code and *bedmap* from the *bedops* tool [65] (v2.4.39) to calculate all these overlaps. See the section “PerSVade pipeline” above for further information on the meaning of each type of SV.

## Supplementary Information

Additional file 1: Figure S1. Detailed workflow of the ‘optimize_parameters’ module. Figure S2. PerSVade’s parameter optimization requires extra resources. Figure S3. PerSVade’s parameter optimization improves the recall of SVs. Figure S4. Global vs SV type-specific parameter optimization. Figure S5. Each sample yields a different set of optimum parameters. Figure S6. PerSVade’s parameters optimization mostly changes the recall of SVs in simulations. Figure S7. Coverage constrains SV calling accuracy in *C. glabrata* simulations. Table S1. Datasets used for the testing in simulations in *C. glabrata*, *C. albicans, C. neoformans, A. thaliana* and *D. melanogaster*.

Additional file 2. Review history.

## Data Availability

PerSVade is available at https://github.com/Gabaldonlab/perSVade [39] and can be installed using either conda environments or through a docker image containing the pipeline, available at https://hub.docker.com/r/mikischikora/persvade. The github repository is released under an open source GNU General Public License (GPL). In addition, the code can be accessed in Zenodo through the DOI 10.5281/zenodo.6866529 [86]. The github repository contains detailed examples on how to install and run perSVade using conda, docker, or singularity. We have tested perSVade on several Linux and Mac architectures, and the docker image may be run in any machine in a reproducible way. All the results shown in this paper were generated using the script https://github.com/Gabaldonlab/perSVade/blob/master/scripts/perSVade.py from version 1.0, which is a wrapper to execute several modules with a single command. Since perSVade is an actively used (and maintained) pipeline, we have created a few new releases since version 1.0, which include an improved documentation, more unit tests, and the implementation of an efficient debugging of inputs. Note that these changes do not affect the functionality of the modules as implemented in version 1.0. Hence, we recommend the usage of the latest version (version 1.02.7 at the time of publication), which is the one with the best documentation and usability. In addition, note that this one-liner wrapper is not recommended for broad usage. All the data used for testing perSVade was obtained from the SRA database or public ftp servers, and is listed in Additional file 1: Table S1 and “Methods.” All the code necessary to reproduce the results and plots shown in this paper is in https://github.com/Gabaldonlab/perSVade/tree/master/testing.
