# Supplementary material for: PerSVade: personalized structural variant detection in any species of interest
Source: Genome Biol. 2022 Aug 16;23:175. doi: 10.1186/s13059-022-02737-4 (PMC9380391; doi:10.1186/s13059-022-02737-4)
Supplement: Supplementary file 1 — Additional file 1: Figure S1. Detailed workflow of the ‘optimize_parameters’ module. Figure S2. PerSVade’s parameter optimization requires extra resources. Figure S3. PerSVade’s parameter optimization improves the recall of SVs. Figure S4. Global vs SV type-specific parameter optimization. Figure S5. Each sample yields a different set of optimum parameters. Figure S6. PerSVade’s parameters optimization mostly changes the recall of SVs in simulations. Figure S7. Coverage constrains SV calling accuracy in C. glabrata simulations. Table S1. Datasets used for the testing in simulations in C. glabrata, C. albicans, C. neoformans, A. thaliana and D. melanogaster. [file 13059_2022_2737_MOESM1_ESM.pdf]

## ADDITIONAL FIGURES

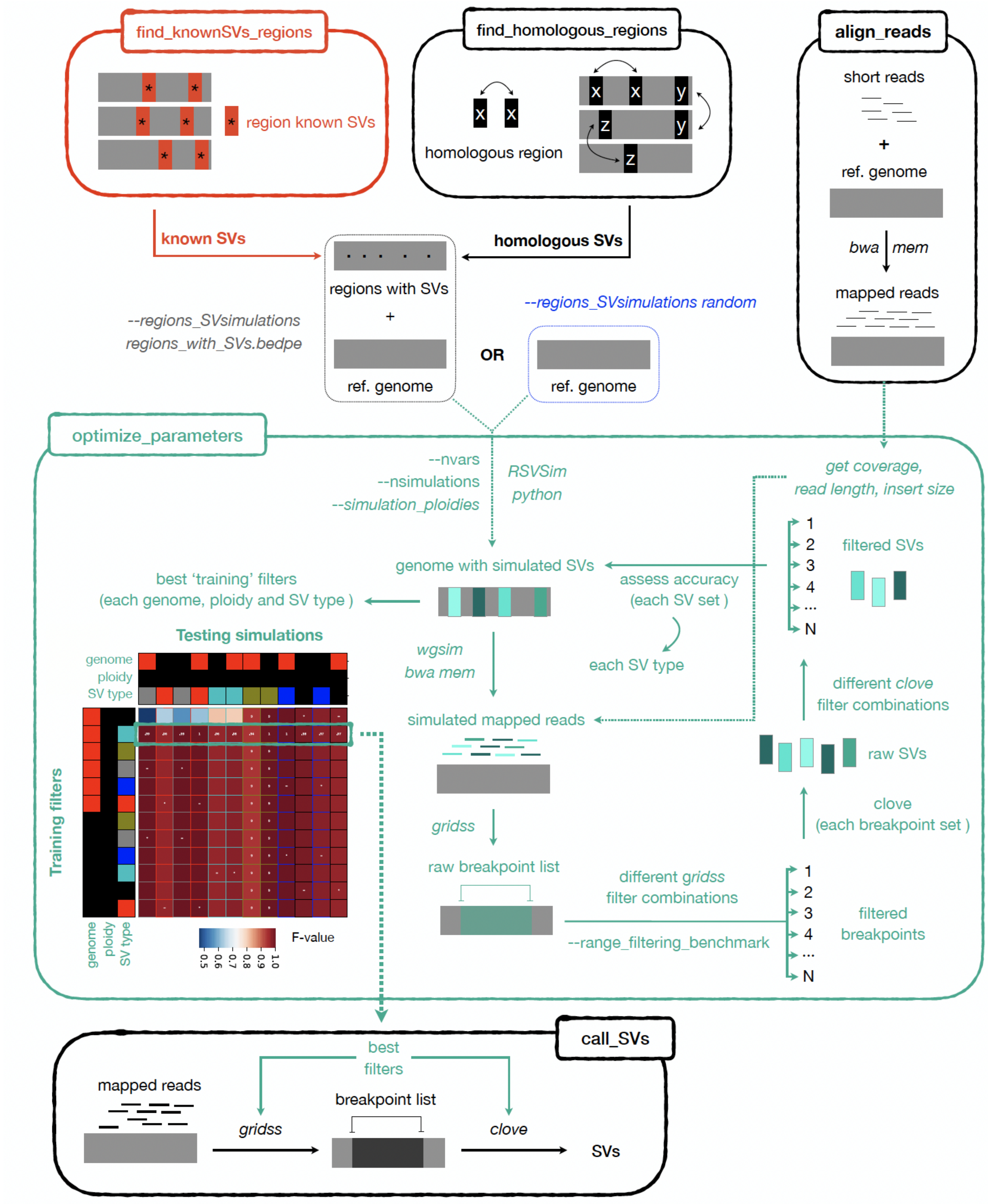


**Figure S1. Detailed workflow of the 'optimize_parameters' module.** The module 'optimize_parameters' is the core, most novel function of perSVade. It requires the argument --regions_SVsimulations, which specifies the regions of the genome for simulations of SVs. These can be either around some specific

regions (with the argument --regions_SVsimulations <regions>.bedpe) or randomly placed across the genome (with the argument --regions_SVsimulations random). Note that perSVade has modules to infer either regions with previously known SVs (through 'find_knownSVs_regions') or regions with pairwise homology (through 'find_homologous_regions'). The SV simulations around such regions may be more realistic than random simulations, which is why they may be considered. The module ‘optimize_parameters’ finds a set of optimum parameters through simulations around these regions. By default, it generates two simulated genomes (tunable with --nsimulations) with 50 SVs of each type (tunable through --nvars) based on the reference genome and the provided regions. There are two simulated genomes for each of the desired ploidies/zygosities, tunable through --simulation_plodies. For example, we set ‘--simulation_plodies haploid’ for haploid organisms and ‘--simulation_plodies diploid_hetero’ for diploids (which means that the simulated genomes will have only heterozygous variants) in the testing of perSVade on several organisms (see **Methods**). For each simulated genome perSVade 'optimize_parameters' simulates reads with equal insert size, coverage and read length as the input mapped reads (provided with the argument -sbam). Then it aligns the reads and runs gridss to obtain a list of 'raw breakpoints'. This module then tries several combinations of filters on them (by default >278,000,000, which is tunable through --range_filtering_benchmark) to generate many 'filtered breakpoints'. Each of these is processed with clove to generate a set of 'raw SVs'. PerSVade ‘optimize_parameters’ next tries several combinations of filters on each of them to get a set of filtered SVs. These are compared against the true set of SVs (inserted in the simulated genome) to calculate the accuracy (F-value) of each combination of *gridss* and clove filters on each simulated genome, ploidy and SV type. These filters are optimized for each simulation, and thus may not be accurate on independent sets of SVs (due to overfitting). In order to reduce this effect, this module tests how each of these 'best' filters perform on all simulations, ploidies and SV types (not only in those that yielded the given filters as optimum). The heatmap shows the F-value for an example sample (BG2 based on random simulations from *Candida glabrata,* see **Methods**), where the filters in the second row are accurate on all simulations (indicating that there is no overfitting on them) and thus they are chosen as the final set of best parameters. Note that the filters in the first row are only accurate on some simulations, suggesting that they are overfitted and thus they are not chosen as good filters. At the end, this module writes the accuracy of these best parameters into a .tab file, which will allow the user to understand how much the results are to be trusted. In addition, these optimized filters (or parameters) are written into a .json file that may be used for calling SVs with 'call_SVs'.

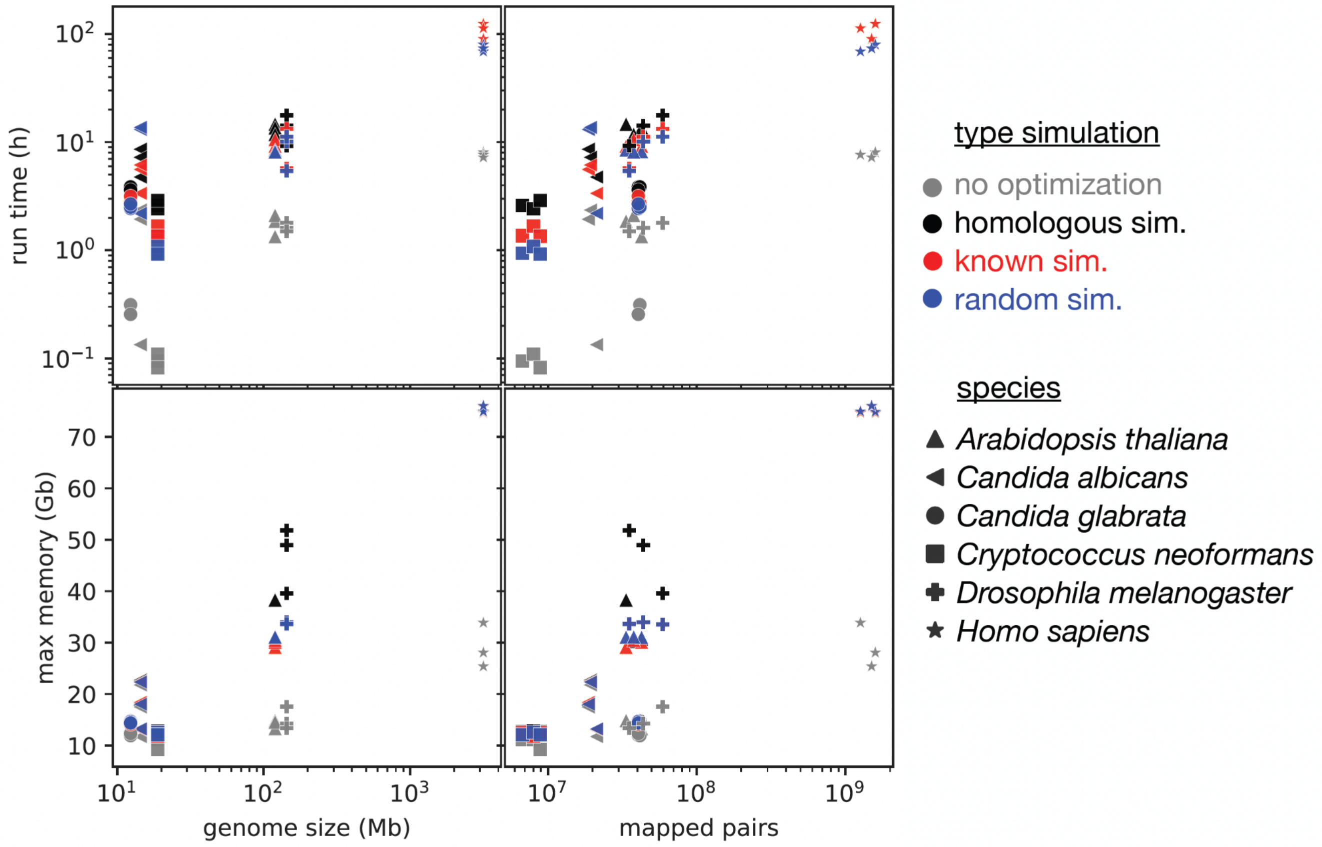


**Figure S2. PerSVade's parameter optimization requires extra resources.** We tested perSVade's SV calling modules ('optimize_parameters', 'call_SVs' and 'integrate_SV_CNV_calls') on six eukaryotes (three samples per species) using either no parameter optimization (gray) or different types of simulations (black, red, blue) for the 'optimize_parameters' module in a machine with 16 cores. Shown are the running time and maximum RAM used ignoring the resources related to read alignment (which was performed independently). Thus, each point reflects the resources used by 'optimize_parameters' (except in the gray points), 'call_SVs' and 'integrate_SV_CNV_calls'. Of note, perSVade was run with a different setting for the human datasets to avoid excessive resource consumption. First, we skipped the marking of duplicate reads on the .bam files. Second, we ran the simulations on a subset of the genome (only chromosomes 2, 7, 9, X, Y and mitochondrial). Third, we skipped the 'homologous' simulations in human samples due to excessive memory consumption. The x axes reflect the reference genome size (left) and the number of mapped read pairs (right), which are correlated with resource consumption. This data may be useful to allocate computational resources for running perSVade.

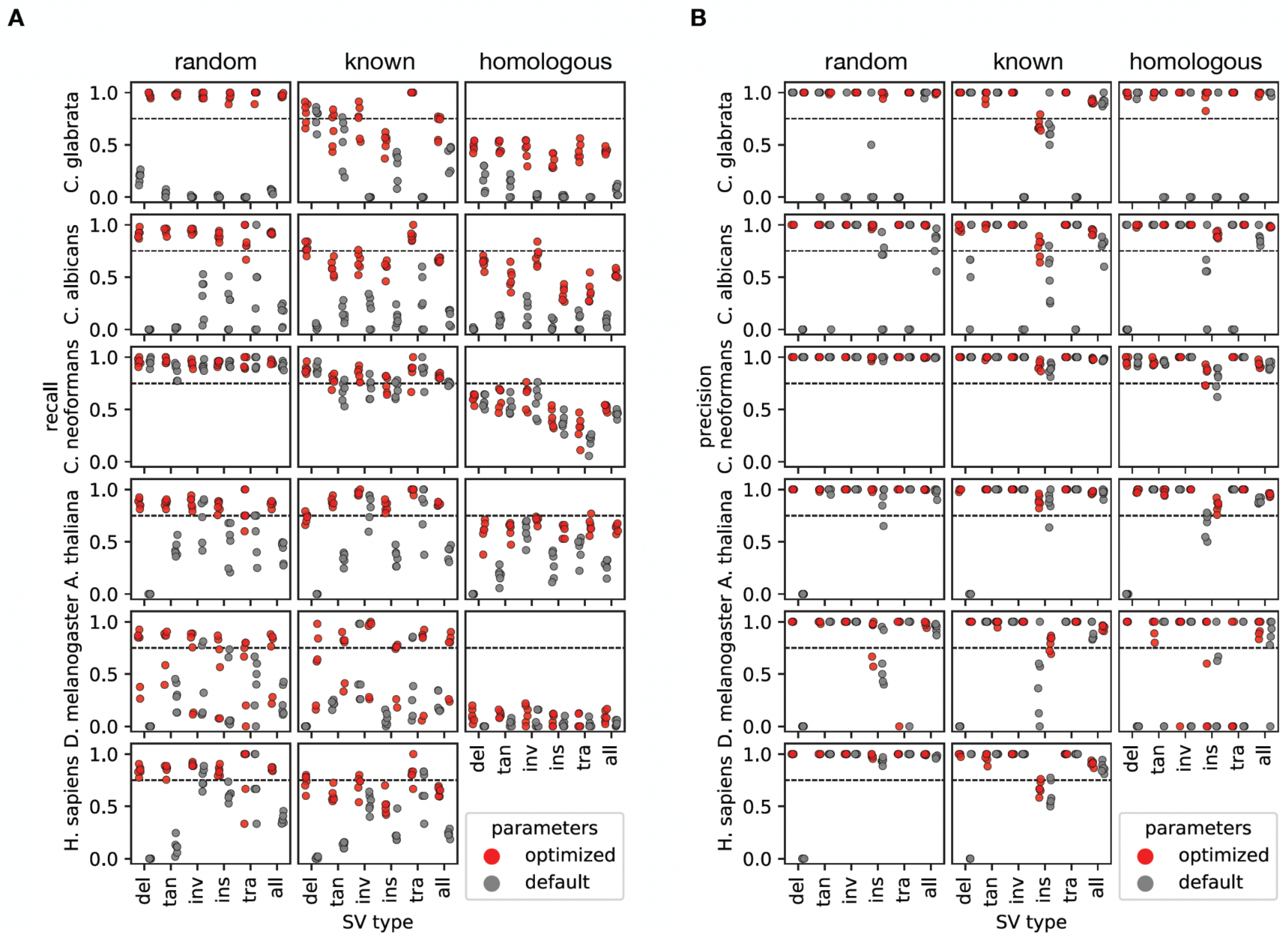


**Figure S3. PerSVade's parameter optimization improves the recall of SVs.** We ran perSVade's SV calling modules on three samples per species for six eukaryotes (see **Methods**) using either 'random', 'known' or 'homologous' simulations. These plots show the recall (left) and precision (right) of either default or optimized parameters (for each sample and simulation type) on these simulations. The x axis represents the type of SV (deletions (del), tandem duplications (tan), inversions (inv), insertions (ins), translocations (tra) and the average of all SVs (all)).

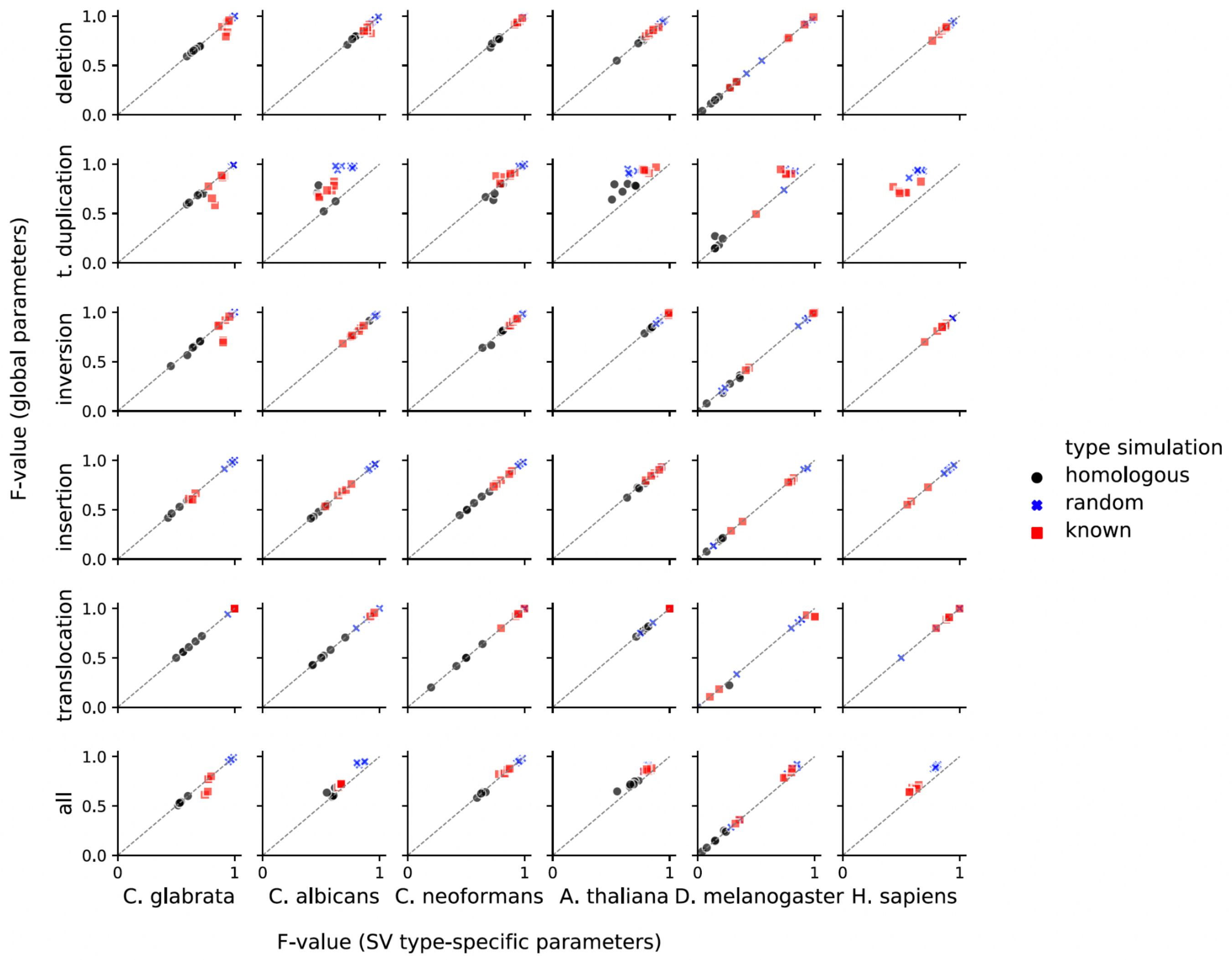


**Figure S4. Global vs SV type-specific parameter optimization.** To understand whether using separate optimal parameters for each SV type is necessary to achieve maximal accuracy we analyzed the intermediate files from the parameter optimization on different species and simulation types (see **Methods**, **Figure 2,3**). Note that perSVade's parameter_optimization module returns a set of parameters that are optimized for all (not only one) simulated genomes and SV types (global parameters). Understanding whether these global parameters are as accurate as those specifically obtained for each simulated genome and SV type is relevant to validate that a single set of parameters can be used for calling all SV types. To find these global parameters, the module first calculates the best parameters for each simulated genome and SV type (SV type-specific parameters) and then tests the accuracy of these parameters on each simulated genome and SV type (see **Additional file 1: Figure S1**). We used these accuracy measurements to understand whether using SV-type specific parameters is more accurate than using global parameters. Each point in these scatter plots represents the SV calling accuracy for a simulated genome considering a given SV type (rows), species (columns) and type of simulations (colors). The x axis represents the calling accuracy (F-value) when using SV type-specific parameters, while the y axis represents the accuracy when using global parameters. Note that we only considered the cases where the global parameters were obtained from a different simulated genome / SV type than the SV-type specific parameters. In addition, note that global parameters perform slightly better than the SV type-specific ones for tandem duplications in some samples of diploid species. This is due to the behavior of the coverage threshold that defines true tandem duplications

(*min_rel_coverage_to_consider_dup* parameter, see **Methods**), where the rationally-designed value set in global parameters (from non-tandem duplication SV types) is more accurate than any of the values tried in the SV type-specific optimization.

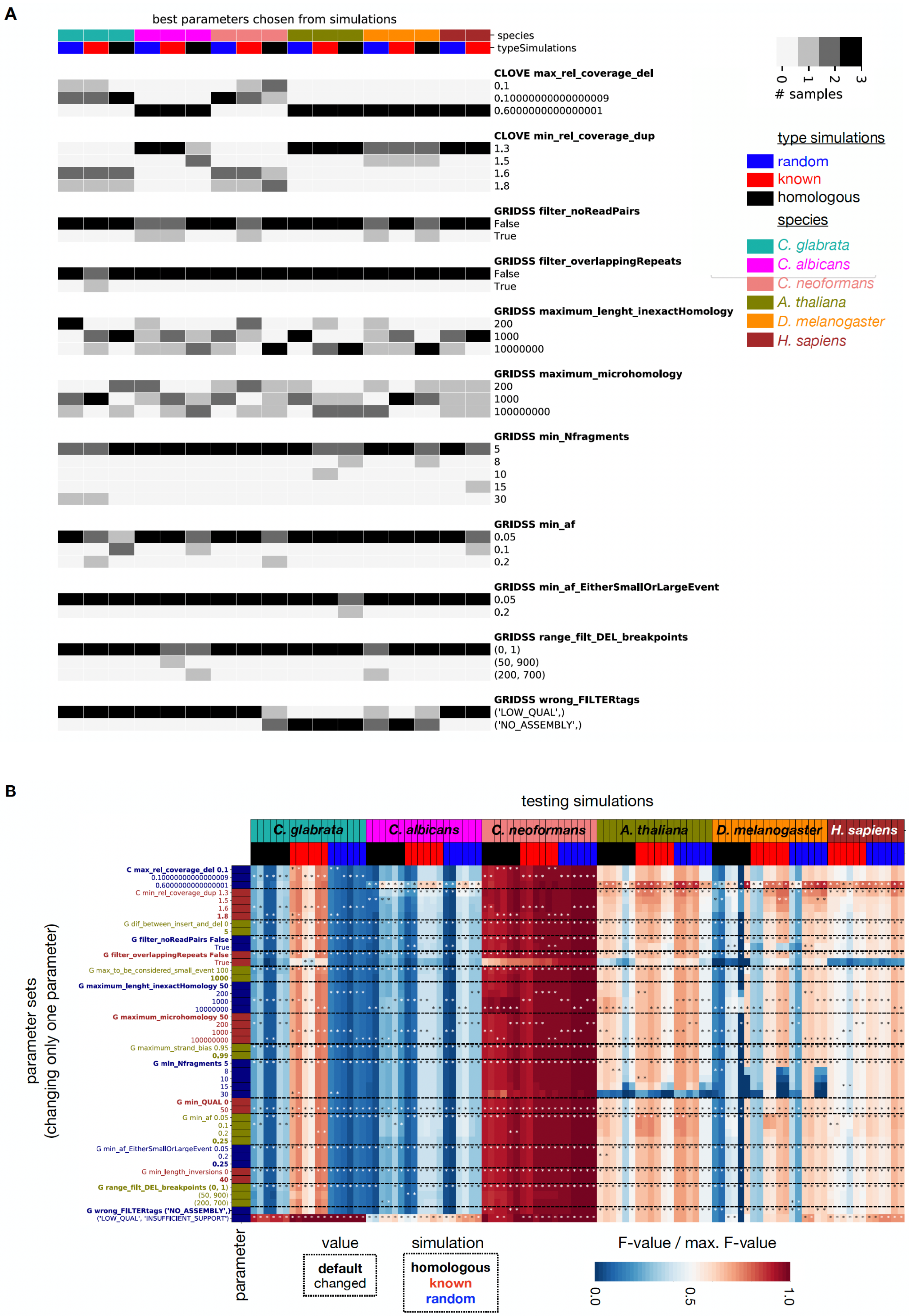
A
best parameters chosen from simulations
species
typeSimulations
CLOVE max_rel_coverage_del
0.1
0.10000000000000009
0.6000000000000001
CLOVE min_rel_coverage_dup
1.3
1.5
1.6
1.8
GRIDSS filter_noReadPairs
False
True
GRIDSS filter_overlappingRepeats
False
True
GRIDSS maximum_lenght_inexactHomology
200
1000
10000000
GRIDSS maximum_microhomology
200
1000
100000000
GRIDSS min_Nfragments
5
8
10
15
30
GRIDSS min_af
0.05
0.1
0.2
GRIDSS min_af_EitherSmallOrLargeEvent
0.05
0.2
GRIDSS range_filt_DEL_breakpoints
(0, 1)
(50, 900)
(200, 700)
GRIDSS wrong_FILTERtags
('LOW_QUAL',)
('NO_ASSEMBLY',)
0
1
2
3
# samples
type simulations
random
known
homologous
species
C. glabrata
C. albicans
C. neoformans
A. thaliana
D. melanogaster
H. sapiens
B
testing simulations
C. glabrata
C. albicans
C. neoformans
A. thaliana
D. melanogaster
H. sapiens
C max_rel_coverage_del 0.1
0.10000000000000009
0.6000000000000001
C min_rel_coverage_dup 1.3
1.5
1.6
1.8
G dif_between_insert_and_del 0
5
G filter_noReadPairs False
True
G filter_overlappingRepeats False
True
G max_to_be_considered_small_event 100
1000
G maximum_lenght_inexactHomology 50
200
1000
10000000
G maximum_microhomology 50
200
1000
100000000
G maximum_strand_bias 0.95
0.99
G min_Nfragments 5
8
10
15
30
G min_QUAL 0
50
G min_af 0.05
0.1
0.2
0.25
G min_af_EitherSmallOrLargeEvent 0.05
0.2
0.25
G min_length_inversions 0
40
G range_filt_DEL_breakpoints (0, 1)
(50, 900)
(200, 700)
G wrong_FILTERtags ('NO_ASSEMBLY',)
('LOW_QUAL', 'INSUFFICIENT_SUPPORT')
parameter sets
(changing only one parameter)
parameter
value
default
changed
simulation
homologous
known
random
F-value / max. F-value
0.0
0.5
1.0

**Figure S5. Each sample yields a different set of optimum parameters. (A)** We ran perSVade's 'optimize_parameters' module on six eukaryotes (three samples per species) and a parameter optimization based on either 'random', 'homologous' or 'known' simulations (see **Methods**). Shown are the chosen values for each parameter (only those that changed across samples) in each optimization procedure. Each group of rows refers to the values chosen for one type of parameter (see **Methods**) used for *gridss* or *clove*. The color indicates how many samples (from zero to three) yielded a specific value for each parameter type. For example, the threshold to discard breakpoints (called by *gridss*) based on allele frequency was set to 0.05 in most samples (see "GRIDSS min_af"). However, the optimization for 'known' SVs in *C. glabrata* yielded a threshold of 0.2 in one sample (out of three) (see the second column). **(B)** To understand the effect of changing each parameter, we measured the SV calling accuracy of different parameter sets, each with only one tuned parameter while all other parameters were kept to default values. We tested all the values obtained in an optimal set (those from A) and the default values. This explains why there are some extra parameters in this panel (*dif_between_insert_and_del*, *max_to_be_considered_small_event, min_QUAL, min_length_inversions* and *min_af_EitherSmallOrLargeEvent*). Each row corresponds to a different parameter set. Each column represents a simulation from a given sample / simulation type to be "tested". The heatmap shows the F-value of each parameter set on each tested simulation, relative to the maximum F-value derived from combinatorial parameter optimization (see **Figure 3A**). Each cell is hereafter referred to as 'testing instance'. The asterisks refer to instances where the changing parameter value is the one picked in the optimization of each sample / simulation type. In addition, the row colors indicate which parameter is tuned in each set. The column color indicates the parameter that is changing (each with a different color), and the fontweight of the label indicates whether the parameter set has default values (labels in bold) or has some parameter value changed. This means that all the rows with default values (bold labels) correspond to the same parameter set. We repeat them for each parameter to aid visual comparisons.

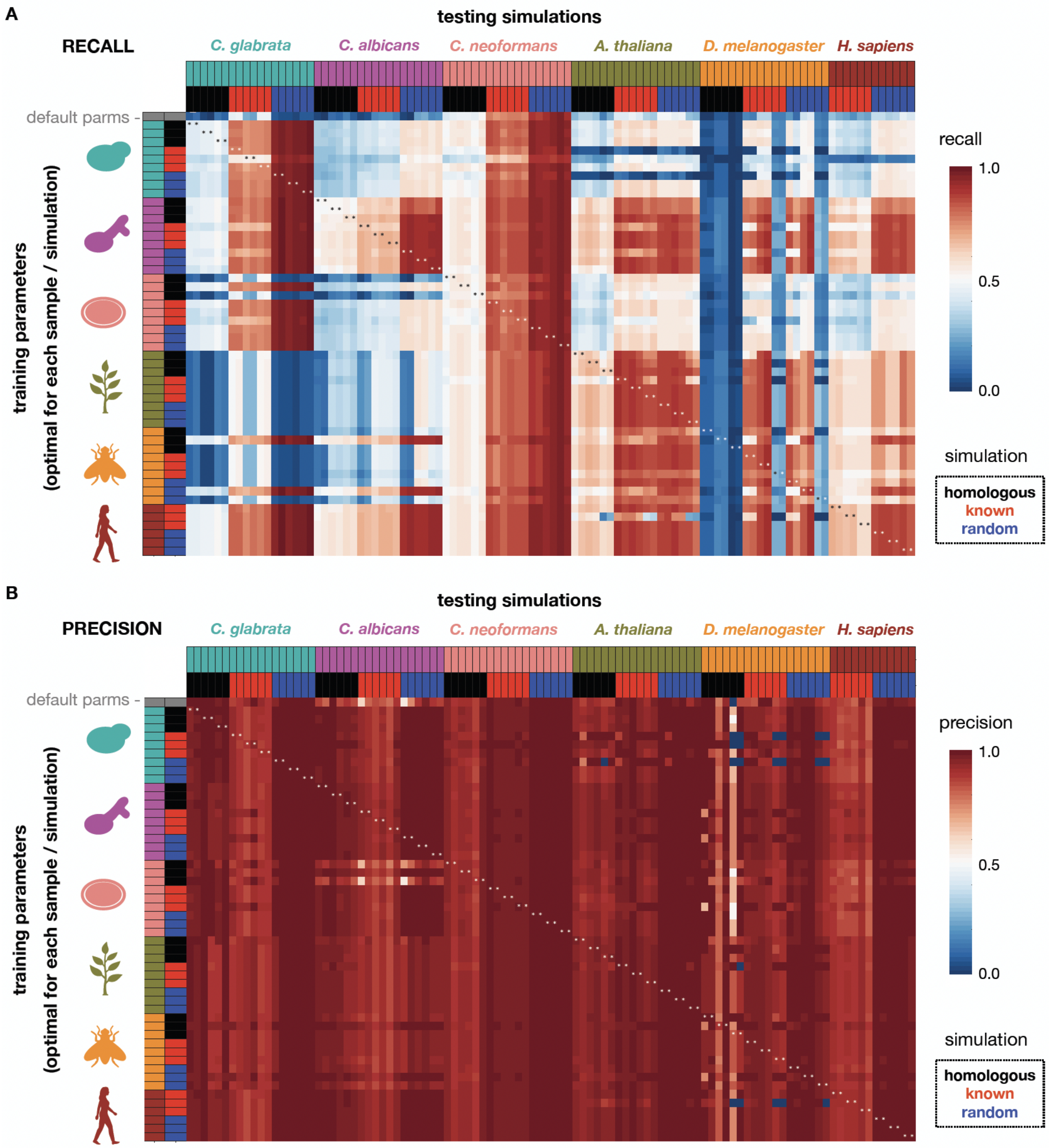


**Figure S6. PerSVade's parameters optimization mostly changes the recall of SVs in simulations.** To assess whether perSVade's parameter optimization is necessary for all samples / simulations (mentioned in **Figure 2 and Additional file 1: Figure S3**) we measured the SV calling accuracy of each parameter set on the other samples / simulations. Each row indicates a different "training" parameter set optimized for each sample and simulation type in all tested species. In addition, the first row refers to the default parameters. Each column represents a simulation from a given sample / simulation type to be tested. The heatmap shows either the recall (A) or the precision (B) of each parameter set on each tested simulation. Note that the species are ordered alike in rows and columns. In addition, note that each sample (from a given species and simulation type) yielded one set of "training" parameters and two simulated genomes tested here, which explains why there are two columns for each row. The asterisks

refer to testing instances where both the sample and type of simulation are equal in the training and testing (equivalent to the ‘optimized’ parameters from **Figure 2 and Additional file 1: Figure S3**).

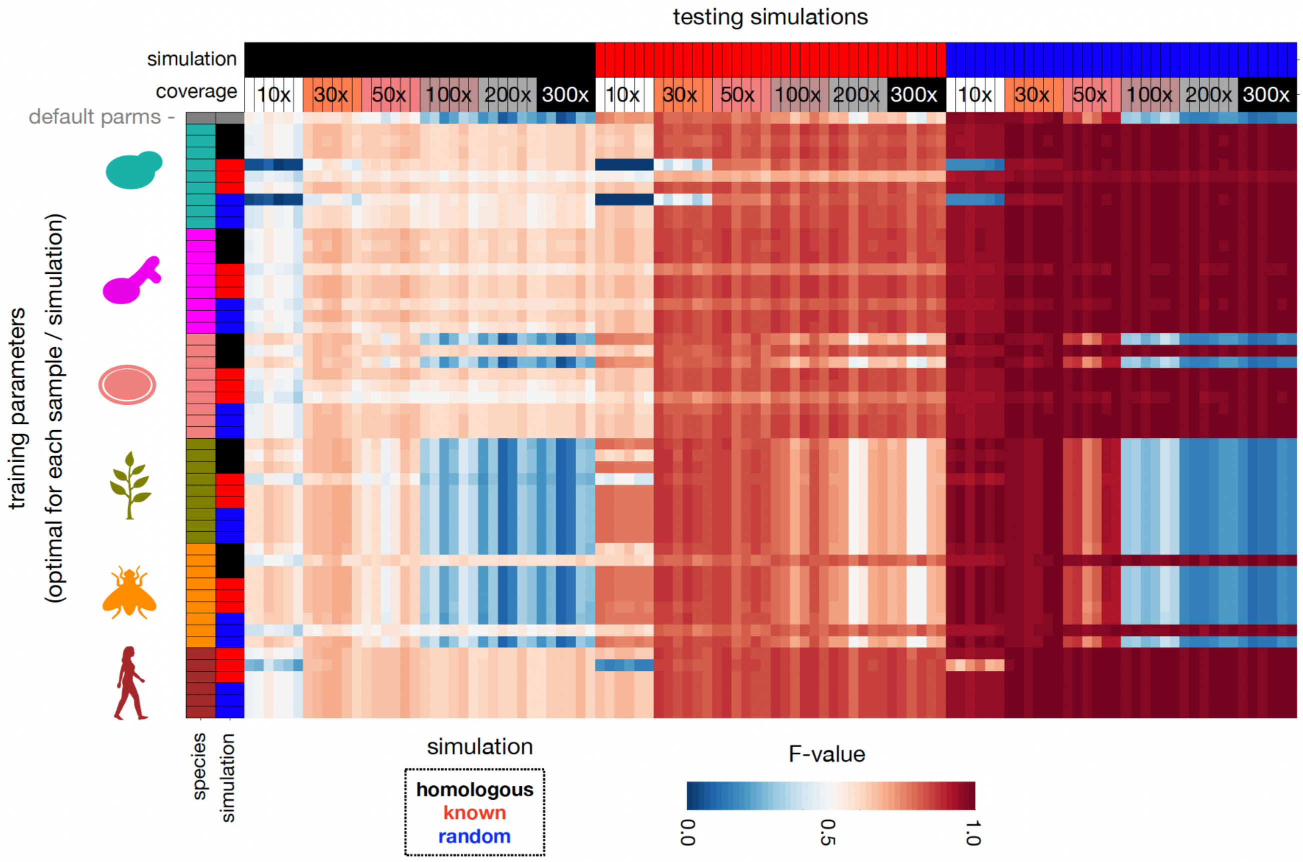


**Figure S7. Coverage constrains SV calling accuracy in *C. glabrata* simulations.** To assess whether the high coverage of *C. glabrata* samples (>300x, see **Table S1**) constrained SV calling, we measured the accuracy of each parameter set (optimized for each species / simulations (see **Figure 3A**)) on the *C. glabrata* simulations with varying coverage. For each simulation (based on a sample and a type of simulation (homologous / known / uniform)), we subsampled randomly the reads to get a coverage of 10x, 30x, 50x, 100x, 200x or 300x. . Each row indicates a different "training" parameter set optimized for each sample and simulation type in all tested species (the same parameters as in **Figure 3A**). In addition, the first row refers to the default parameters. Each column represents a simulation from a given sample / simulation type / coverage to be tested. The heatmap shows the F-value of each parameter set on each tested simulation. Note that the species symbols correspond to *C. glabrata*, *C. albicans*, *C. neoformans*, *A. thaliana, D. melanogaster* and *H. sapiens*, respectively. Note that the cells related to 300x coverage are similar to the original simulations (**Figure 3A**).

## ADDITIONAL TABLES

| target_species | target_taxID | sample_taxID | sample_species | SRA_run | % reads map. | cov. |
|---|---|---|---|---|---|---|
| *C. glabrata* | N/A | N/A | *C. glabrata BG2* | SRR15498429 | N/A | 319 |
| *C. glabrata* | N/A | N/A | *C. glabrata* CST34 | SRR15498440 | N/A | 342 |
| *C. glabrata* | N/A | N/A | *C. glabrata M12* | SRR15498481 | N/A | 337 |
| *C. albicans* | 5476 | 1182531 | *C. albicans 3153* | SRR641729 | 94.58 | 128 |
| *C. albicans* | 5476 | 1182537 | *C. albicans A123* | SRR538772 | 88.77 | 89 |
| *C. albicans* | 5476 | 1182540 | *C. albicans A203* | SRR538786 | 95.91 | 92 |
| *C. neoformans* | 5207 | 1423894 | *C. neoformans Bt35* | SRR1063293 | 99.93 | 30 |
| *C. neoformans* | 5207 | 1423915 | *C. neoformans RSA-MW-1281* | SRR1063017 | 99.86 | 37 |
| *C. neoformans* | 5207 | 1423916 | *C. neoformans RSA-MW-5465* | SRR1063214 | 99.94 | 40 |
| *A. thaliana* | 3702 | 38785 | *A. arenosa* | SRR4128971 | 76.76 | 22 |
| *A. thaliana* | 3702 | 378006 | *A. arenosa x A. thaliana* | ERR5032500 | 89.59 | 25 |
| *A. thaliana* | 3702 | 2608267 | *A. arenosa x A. lyrata* | ERR3514861 | 65.98 | 21 |
| *D. melanogaster* | 7227 | 7238 | *D. sechellia* | SRR5860659 | 89.70 | 37 |
| *D. melanogaster* | 7227 | 7240 | *D. simulans* | ERR1597900 | 84.44 | 45 |
| *D. melanogaster* | 7227 | 7243 | *D. teissieri* | SRR13202235 | 86.61 | 6 |

**Table S1. Datasets used for the testing in simulations in C. glabrata, *C. albicans, C. neoformans, A. thaliana* and *D. melanogaster*.** We chose these datasets automatically from the SRA database for *C. albicans, C. neoformans, A. thaliana* and *D. melanogaster*. In order to have enough SV calls we selected mildly divergent samples (as compared to the reference genome) with a NCBI taxonomy taxon ID (indicated by each sample_taxID) different from the ID of species of interest (target_taxID). However, we only kept samples with most reads mapped (specified in the column '% reads map.') in order to discard datasets from highly divergent taxa. Note that it was not possible to find such samples for *C. glabrata* at the time of this study. We thus used three datasets for *C. glabrata* strains from our lab (see **Methods**). 'N/A' indicates that the column (i.e. taxID or % of mapped reads) was not taken into consideration for selecting these samples. See **Methods** for more information. Note that the 'cov.' column indicates the read depth of each of these samples.
