# Supplementary material for: PerSVade: personalized structural variant detection in any species of interest
Source: Genome Biol. 2022 Aug 16;23:175. doi: 10.1186/s13059-022-02737-4 (PMC9380391; doi:10.1186/s13059-022-02737-4)
Supplement: Supplementary file 2 — Additional file 2. Review history. [file 13059_2022_2737_MOESM2_ESM.docx]

Review History

**First round of review**

**Reviewer 1**

**Were you able to assess all statistics in the manuscript, including the appropriateness of statistical tests used?** Yes, and I have assessed the statistics in my report.

**Were you able to directly test the methods?** No.

**Comments to author:**

Schikora-Tamarit and Gabaldón present PerSVade, a SV calling pipeline built around the existing GRIDSS SV calling tool. The paper is well-written and easy to follow but leaves me with mixed feelings. On a positive note, the manuscript is an eye-opener that SV calling from short reads is still a challenging task and that practitioners should thread with care when choosing a tool and a set of parameters. On the other hand, the manuscript leaves me with a number of questions.

1) This empirical study reveals (very) large differences in F1-score due to different choices of parameters. Even though the parameters themselves are listed in supplementary material, there is little insight into why the default set of parameters is suboptimal (or even downright inappropriate) for certain species/datasets. For example, for C. glabrata (only ~12.3 Mb in size), performance with default parameters appears very low (F-score < 0.1) whereas this seems to improves to >0.9 after tuning. Some insights would be appreciated: is there a limited number of key parameters that need to be tuned (and if so, what parameter?) or it is really a complex interplay between many different parameters?
2) Most performance metrics are summarized into F1-scores. From Fig. 4, it appears that the default parameters suffer largely from low recall (at least for human). In other words, one or more of the default parameters may be set too stringent/conservative. Is this a general observation?
3) I understand from the manuscript that PerSVade comes with a very large computational burden, especially for larger genomes such as the human genome. Nevertheless, I find it difficult to relate the substantial accuracy gains obtained through PerSVade (also in human) with the statement that PerSVade is primarily intended for smaller genomes?
4) Even though the authors argue that a comparison to existing tools (e.g. DELLY or LUMPY) is outside the scope of their paper, the lack of such comparison leaves the reader with the inevitable question: can PerSVade also be applied to other SV callers and if so, does it promise the same gains in accuracy?

Minor
1) This is probably a matter of taste, but to me, the word "personal" refers to an "individual", rather than a species.
2) Is GRIDSS or GRIDSS2 used? Both are referenced.

**Reviewer 2**

**Were you able to assess all statistics in the manuscript, including the appropriateness of statistical tests used?** Yes, and I have assessed the statistics in my report.

**Were you able to directly test the methods?** Yes.

**Comments to author:**

The authors present the tool perSVade which in general in designed in order to automatically optimise the parameters of certain short-read SV detection tools in order to improve accuracy. I am very fond of the idea of a tool which can optimise parameters automatically to fit any species considering most tools are tailored-towards/designed-in a species of interest and show varying results (generally worse) within different genomic backgrounds. I would add, on top of what the authors have written, that the 'one-size-fits-all' method is not only sub-optimal but in fact biased towards certain species. The results with perSVade appear conclusive that species focussed parameter optimisation of tools will help in most cases, highlighting both the above issues and the utility of the tool.
I have a few comments/questions below but in general the tool seems well thought out, from pre-processing to output format, and should be very welcomed in the field of short read SV detection which continues to have utility.

1. Page 6 line 34, it appears here you are saying that inversions are represented by a single breakpoint/line? Or if I understand correctly from your github, both breakpoints for an inversion have a single line each however each line also contains the information of both the start and end of the inversion as well?

2. Although I see the reasons for selecting solely gridss as the caller, I am wondering if you have run similar optimisation modules on other SV callers?
Similarly, as I understand the optimisation paramaters impact only gridss SV calling, but have no impact on the tools used in the 'call_CNV' module. Is that the case and if so, why?

3. I am unsure as to whether de-novo insertions are called or not. Page 6 line 48, says that 'perSVade' the tool does not call de-novo insertions when describing the 'call_SV' module, however, pg9 line57 mentions de-novo insertions when merging 'call_SV' and 'call_CNV'.
Perhaps de-novo insertions are only called within the 'call_CNV' module? This seems doubtful considering this is determined by coverage analysis. So essentially, as above, I am confused as to whether they are actually called or not.
If this is a mistake and de-novo insertions are never called by perSVade, why is this the case? Only because of the 'annotate_SVs' module?

4. I am not sure I completely understand the simulation step. Page 7 line 7, says 50 SVs of each type are generated in 2 simulated genomes.
Why 2 different genomes? Is this because the default is diploid?
Does using 50 SVs per genomes regardless of size impact the outcome? Should larger genomes perhaps contain more simulated SVs and smaller fewer? (I understand this can be changed using the -nvars option)

5. For 'diploid heterozygous' genomes, why do you make all simulated SVs heterozygous? Shouldn't some be homozygous still?
Could you make it obvious what other options are available with the -simulation_ploidies option? For example, are triploid_homo or tetraploid_hetero appropriate input values? Perhaps it would be easier with just a number for the ploidy (as with the call_CNV module although I see it is limited to 1 or 2?) and an option for zygosity (homo/hetero) so that all combinations are possible?

6. Page 7 line 38, you mention how the optimisation is in order to call all SV types. Have you tried splitting it in order to find the optimum parameters per SV type? And if so, does it help much?
If this was added, it could perhaps be indicated to be a 'slow but accurate' option (assuming it is more accurate).

7. The modules to find repetitive regions and use this information during SV simulation is a very nice addition. It is also great to see how the impaired results of whilst using this step during simulation could perhaps explain the discrepancy between simulated and real datasets, in that SVs are commonly found in repetitive regions.

8. Testing across samples and species nicely shows how each genome can require a very different set of parameters. Could you perhaps give some details as to which parameters are most variable among different species and if these parameters/differences may say something about the genomic background? This could also be done for test with and without the consideration of homologous regions/using real data. This would perhaps only be interesting if few parameters contained most of the variability.

9. Page 9 line 46, How exactly is the redundancy defined between calls from the 'call_SV' and 'call_CNV' modules?

10. 1Page 12 line 40, did you forget to mention the use of the 'call_CNV' module?

11. Could you add a 'fast' option to speed up the process for large genomes? What process takes up the most of the time? Could the parameter optimising steps be run on just half or a subset of the chromosomes (as performed for the human genomes, Page 13 line 7)?

12. Is there some idea as to why there is little improvement in the detection of SVs in C. neoformans?
Is there any example where it gets worse and in the case that the defaults are the optimum can it make no changes to the parameters?

13. Using the tool requires the use of many different modules each with their own output which in turn needs to be fed into the next module. Would you be able to provide an option in which the tool does it all in one go (trimming, aligning, optimising, calling SVs/CNVs, merging, etc)? It seems there was already this function previously but you are removing this feature?

14. The FAQs page on the github is rather detailed and clarifies simple questions that arise during the process of running the modules.

**Authors Response**

**Point-by-point responses to the reviewers’ comments:**

Reviewer reports:

Reviewer #1: Schikora-Tamarit and Gabaldón present PerSVade, a SV calling pipeline built around the existing GRIDSS SV calling tool. The paper is well-written and easy to follow but leaves me with mixed feelings. On a positive note, the manuscript is an eye-opener that SV calling from short reads is still a challenging task and that practitioners should thread with care when choosing a tool and a set of parameters. On the other hand, the manuscript leaves me with a number of questions.

**Response:** We thank reviewer 1 for his positive comments and the constructive remarks.

1) This empirical study reveals (very) large differences in F1-score due to different choices of parameters. Even though the parameters themselves are listed in supplementary material, there is little insight into why the default set of parameters is suboptimal (or even downright inappropriate) for certain species/datasets. For example, for C. glabrata (only ~12.3 Mb in size), performance with default parameters appears very low (F-score < 0.1) whereas this seems to improves to >0.9 after tuning. Some insights would be appreciated: is there a limited number of key parameters that need to be tuned (and if so, what parameter?) or it is really a complex interplay between many different parameters?

**Response:** We agree that this is a relevant question. In order to achieve reasonable performance, perSVade’s parameter_optimization module includes a step to reduce the number of parameter combinations to be optimized. Starting from a range of values assigned to each parameter (customizable through the option *--range_filtering_benchmark*) our pipeline discards values that do not change breakpoint filtering as compared to an unconservative set of parameters. This means that the set of parameters tuned in each optimization run are already limited to those that can be relevant, and these could be different for each species or sample. This is now clarified in the methods section.

Inspired by the reviewer’s question we did some additional analyses to understand why the default set of parameters is suboptimal. For this, we analyzed how changing each parameter in isolation (while keeping all other parameters in default values) affects accuracy in different species and types of simulations. These results are shown in the new Figure S5B and we believe that they provide significant insights that address the reviewer’s question. In general, we find that the accuracy reached by changing single parameters is mostly below the accuracy reached from combinatorial parameter optimization. This suggests that there is often a complex interplay between different parameters, which justifies the need for such optimization. However, there are some parameters that can have a huge impact independently of the others. We now report the effect of different parameters in the revised manuscript. This explains why the default parameters are suboptimal.

The example of *C. glabrata*, mentioned by the reviewer, is actually very interesting. We find that the default value for the *gridss* parameter ‘wrong_FILTERtags’ (called ‘NO_ASSEMBLY’, which filters out breakpoints that have no *de novo* assembly evidence) yields low accuracy in *C. glabrata* simulations. Changing this parameter to ‘LOW_QUAL, INSUFFICIENT_SUPPORT’ (which only filters out breakpoints that have low quality or low support) is enough to reach very high accuracy, close to the optimal accuracy obtained with combinatorial optimization. Interestingly, this effect is not observed in the other species, which further justifies the need for species-tailored filtering of SVs. We report this and similar findings in the revised manuscript. Different intrinsic genomic characteristics of each species, or technical differences in sequencing runs may explain these intricacies and highlight the usefulness of the optimization phase.

2) Most performance metrics are summarized into F1-scores. From Fig. 4, it appears that the default parameters suffer largely from low recall (at least for human). In other words, one or more of the default parameters may be set too stringent/conservative. Is this a general observation?

**Response:** Yes, this is a general observation also for other species (see Figures S3 and S6). Using the analysis described above (see Figure S5B) we find that default parameters are too conservative for many species, largely because of the strategy to filter based on the FILTER vcf column and the coverage thresholds used for tandem duplications and deletions. This has been clarified in the results.

Note that the default parameters were designed as a baseline to understand the need for parameter optimization. We thus pre-defined these parameters in a standard way: based on author recommendations (from previous filtering strategies designed by the *gridss* authors in human genomes). We think that this *a priori* definition of default parameters, not influenced by the benchmarking results, was essential to have a true baseline (choosing parameters based on author recommendations). This also means (and more even so after considering the results of our benchmarking) that these default parameters may not be optimal for most species, and we thus recommend parameter optimization. This has been clarified in the methods section.

3) I understand from the manuscript that PerSVade comes with a very large computational burden, especially for larger genomes such as the human genome. Nevertheless, I find it difficult to relate the substantial accuracy gains obtained through PerSVade (also in human) with the statement that PerSVade is primarily intended for smaller genomes?

We agree that the accuracy improvement in larger genomes may justify the use of perSVade on them. However, we also think that the computational burden of parameter optimization (see Figure S2) could be too high for some users, and it is a limitation that should be mentioned in the manuscript. We consider that the chosen settings should be a matter of user choice, depending on available resources. We have clarified this tradeoff in the manuscript. In addition, we propose a (recommended) cost-effective strategy for different samples with large genomes: running parameter optimization for only one sample and then using the optimized parameters for all other samples.

4) Even though the authors argue that a comparison to existing tools (e.g. DELLY or LUMPY) is outside the scope of their paper, the lack of such comparison leaves the reader with the inevitable question: can PerSVade also be applied to other SV callers and if so, does it promise the same gains in accuracy?

**Response:** We agree that this is a relevant question. The fact that filtering strategies affect the SV calling accuracy has been reported for several algorithms. For example, Kosugi et. al. 2019 show how filtering SVs (called by DELLY, forestSV, Pindel and BASIL-ANISE) based on varying numbers of supporting reads affects the accuracy (see Figure S2 in their paper). Similarly, the benchmarking of 10 SV callers (including manta and gridss) reported in Cameron et. al. 2019 reflects that changing the accepted FILTER tags from the vcf file or the required minimum quality largely constrains the calling accuracy. These observations suggest that most SV callers could be boosted with a parameter optimization strategy such as the one described in perSVade for *gridss* and *clove*. We have added this discussion to the paper.

With respect to the question “can perSVade also be applied to other SV callers?”, the discussion above suggests optimization is likely to benefit most callers. However, given the different approaches, formats and parameters used for each caller, each caller would need a specific implementation of such optimisation. Currently, perSVade is intimately adapted to the output formats, types of SVs and filtering parameters of *gridss* and *clove*. There are several reasons why re-implementing perSVade for other callers is not a trivial task. Various SV callers output diverse types of SVs and require very different sets of filtering parameters. A perSVade-like pipeline based on other callers would require developing modules like ‘call_SVs’, ‘integrate_SV_CNV_calls’, ‘annotate_SVs’ and ‘optimize_parameters’ adapted to each algorithm. Note that the current modules are based on thousands of lines of custom code specifically designed for *gridss* and *clove*. We thus consider that it is almost impossible to develop a pipeline that is adapted to any SV caller due to this high heterogeneity in output formats and types of SVs. Alternatively, we could develop new perSVade modules to run SV calling based on particular alternative callers, which is definitely an interesting follow up project. However, this would require rewriting most of the code from scratch, adapting them to each of the callers. Considering that these modules span >10,000 lines of code, we think that this is out of the scope of this manuscript. We consider that our approach (focused only on *gridss* and *clove*) is appropriate given the recommendation of using a single, high-performing algorithm for breakpoint calling instead of multiple software (see Cameron et. al. 2019). We have added these considerations to the discussion.

Minor

1) This is probably a matter of taste, but to me, the word "personal" refers to an "individual", rather than a species.

**Response:** We agree that this could lead to misinterpretation. However, note that perSVade adapts the parameters to any individual of any species, which in fact justifies the usage of ‘personalized’. We have clarified this in the introduction. In addition, note that perSVade already has several users and it was presented in some conferences. We think that changing the name could generate confusion among current and future users, which is why we would rather not do it. We hope the reviewer understands this.

2) Is GRIDSS or GRIDSS2 used? Both are referenced.

**Response:** We used *gridss* 2 in perSVade. However, we designed the default parameters also following advice reported in the original *gridss* paper. The references are now corrected to reflect this.

Reviewer #2: The authors present the tool perSVade which in general in designed in order to automatically optimise the parameters of certain short-read SV detection tools in order to improve accuracy. I am very fond of the idea of a tool which can optimise parameters automatically to fit any species considering most tools are tailored-towards/designed-in a species of interest and show varying results (generally worse) within different genomic backgrounds. I would add, on top of what the authors have written, that the 'one-size-fits-all' method is not only sub-optimal but in fact biased towards certain species. The results with perSVade appear conclusive that species focussed parameter optimisation of tools will help in most cases, highlighting both the above issues and the utility of the tool.

I have a few comments/questions below but in general the tool seems well thought out, from pre-processing to output format, and should be very welcomed in the field of short read SV detection which continues to have utility.

**Response:** We thank the reviewer for the positive considerations about our work. We have added in the discussion that the typical 'one-size-fits-all' method is likely biased towards certain species.

1. Page 6 line 34, it appears here you are saying that inversions are represented by a single breakpoint/line? Or if I understand correctly from your github, both breakpoints for an inversion have a single line each however each line also contains the information of both the start and end of the inversion as well?

**Response:** We agree that this is confusing and not properly explained in the text. PerSVade has two types of outputs. The module ‘call_SVs’ generates one tabular file for each type of SV (deletions, inversions, tandem duplications, insertions, translocations and unclassified breakpoints), where each line corresponds to one variant. For example, each inversion is written into a line of the ‘inversions.tab’ file. This is independent of the number of breakpoints that are related to each SV. For example, each inversion is related to two breakpoints (following the *clove* classification). This has been clarified in the methods section describing the ‘call_SVs’ module.

In addition, the module ‘integrate_SV_CNV_calls’ integrates all the variants into a single vcf file showing how SVs alter specific genomic regions. We designed this vcf to be compatible with the Variant Effect Predictor (VEP) tool for functional annotation, which can interpret tandem duplication (TDUP) duplication (DUP), deletion (DEL) and breakend-like (BND) events. This required the decomposition of each variant into such TDUP, DUP, DEL, BND events (one event in each row of the vcf). The FAQs section in [https://github.com/Gabaldonlab/perSVade/wiki/8.-FAQs](https://github.com/Gabaldonlab/perSVade/wiki/8.-FAQs#what-is-in-sv_and_cnv_variant_callingvcf) explains precisely how we did this decomposition. In the case of inversions we decomposed them into two BND events (two rows in the vcf), one for each end of the inversion. The rationale behind this is that, in terms of functional annotation for inversions, we are interested in genomic features that are around the ends of the inversion, where the rearrangement happens. We have clarified in the methods section that the design of this vcf file is related to VEP compatibility.

2. Although I see the reasons for selecting solely gridss as the caller, I am wondering if you have run similar optimisation modules on other SV callers? Similarly, as I understand the optimisation paramaters impact only gridss SV calling, but have no impact on the tools used in the 'call_CNV' module. Is that the case and if so, why?

**Response:** Regarding the optimisation modules for other SV callers, please see our response to point 4 of reviewer 1 above.

As for the impacts of optimisation parameters. Yes, indeed, this is the case, and we have clarified it in the methods section. The ‘call_SVs’ and ‘call_CNVs’ modules use fundamentally different algorithms. This means that the filtering parameters for SV calling would not be useful for coverage-based CNV calling. A parameter optimization module for CNV calling could be an interesting addition to the pipeline, but it would be a challenging project in itself. We consider that this could be particularly challenging because the outputs of the CNV calling algorithms (HMMcopy, CONY or AneuFinder) do not include confidence attributes (such as variant quality or FILTER vcf tags) that may allow parameter optimization as we do with SV calling. We believe that implementing such a parameter optimization is out of the scope of this manuscript.

Note that, we designed the CNV calling module as a complement to perSVade, allowing for the identification of several types of variants in the same framework. However, this module is just a (standard) implementation of available software, without much scientific novelty. This is why this work is centered on the (actually novel) SV calling features of perSVade.

3. I am unsure as to whether de-novo insertions are called or not. Page 6 line 48, says that 'perSVade' the tool does not call de-novo insertions when describing the 'call_SV' module, however, pg9 line57 mentions de-novo insertions when merging 'call_SV' and 'call_CNV'.

Perhaps de-novo insertions are only called within the 'call_CNV' module? This seems doubtful considering this is determined by coverage analysis. So essentially, as above, I am confused as to whether they are actually called or not.

If this is a mistake and de-novo insertions are never called by perSVade, why is this the case? Only because of the 'annotate_SVs' module?

**Response:** We agree that this is confusing and needs clarification. *Gridss* reports two types of *de novo* insertions: non-template insertions found around the breakpoints and also actual *de novo* insertions (unrelated to a breakpoint). However, the usage of short reads limits the calling of actual *de novo* insertions to small events, which may miss many real *de novo* insertions. This is why we decided to not consider *de novo* insertions as truthful events in the ‘optimize_parameters’ and ‘call_SVs’ modules. However, since perSVade saves the *gridss* output the unfiltered small *de novo* insertions can be obtained (although these should be treated with great caution). We have clarified this in the methods section.

Conversely, the non-template insertions around breakpoints are likely to be the product of DNA repair after a rearrangement (i.e. an inversion or translocation). We believe that these insertions are interesting in terms of functional annotation, since they can modulate the impact of SVs on genomic features. This is why the integrated vcf generated by ‘integrate_SV_CNV_calls’ (designed for functional annotation) also reports them. We have clarified this in the methods.

4. I am not sure I completely understand the simulation step. Page 7 line 7, says 50 SVs of each type are generated in 2 simulated genomes.

Why 2 different genomes? Is this because the default is diploid?

**Response:** We apologize for the lack of clarity. By default, the module ‘optimize_parameters’ generates two simulated genomes (based on the reference genome) with 50 SVs of each type. The ploidy is unrelated to the number of simulated genomes. In fact, the ploidy is taken into account through the *--simulation_ploidies* parameter, which indicates the zygosity and ploidy. For example, using ‘*--simulation_ploidies diploid_heterozygous --nsimulations 2*’ (this is what we did in the benchmarking for diploid organisms) generates two simulated genomes, each with heterozygous SVs. There is no default value for --*simulation_ploidies*. We have clarified this in the methods section.

We think that it is important to have at least two simulated genomes (each with unique sets of SVs) to make sure that the optimized parameters are not overfit to one particular simulated genome. In the former example for diploid organisms, the module ‘optimize_parameters’ first finds a set of parameters that are optimal for each SV type in each simulated genome (training parameters), and then measures the accuracy of each of these ‘optimal parameters’ on the other types of SVs and simulated genomes (testing SV sets). We show an example of this ’overfitting evaluation’ in Figure S1, where most of the training parameters yield high accuracy on the testing sets. There is this interesting exception of the training parameters of the first row (optimized for deletions in the first simulated genome), which are inaccurate in many test sets and are likely overfitted. We have clarified this in the methods section.

Does using 50 SVs per genomes regardless of size impact the outcome? Should larger genomes perhaps contain more simulated SVs and smaller fewer? (I understand this can be changed using the -nvars option)

**Response:** We agree that this is an interesting question. We could not find clear evidence on the average expected number of SVs in different individuals of all the tested species, so that the setting of the number of simulated variants was not trivial. We consider that setting a realistic number of SVs is not necessarily only related to genome size, as the average (populational) divergence to the reference genome or the rate of SV formation could be equally important. In addition, increasing the number of variants heavily influences the computational needs of parameter optimization. We found that 50 variants of each type was a reasonable tradeoff to ensure meaningful calculations of accuracy without a too high resource burden. As implemented now, the variant size is correlated with genome size, which seems a reasonable way to ensure that long genomes have larger sections under SV. We consider that this already fits the suggestion made by the reviewer. We have clarified this in the methods.

In general, as mentioned by the reviewer, each user can change this number through the *--nvars* argument, allowing the simulation of genomes with realistic numbers of variants. It is likely that users want to tune this parameter to resemble the samples of interest (depending on intraspecific diversity and mechanisms of SV formation in a given species) and the available resources. We have clarified in the methods that it could be essential to tune this parameter to the user’s needs.

5. For 'diploid heterozygous' genomes, why do you make all simulated SVs heterozygous? Shouldn't some be homozygous still?

**Response:** In fact, this is a tunable option through --simulation_ploidies, and we agree that it is not clear in the current manuscript. We have clarified in the methods how to obtain parameters optimized for different ploidies / zygosisties.

In our testing of the pipeline on diploid species we decided to only simulate diploid heterozygous variants to create the most challenging scenario for SV calling (since homozygous variants are expected to be easier to find due to higher coverage). A similar strategy was used in a recent benchmarking study (Cameron et. al. 2019), suggesting that it is a valid method to understand SV calling accuracy. We have clarified the reasons for our choice in the methods.

Could you make it obvious what other options are available with the -simulation_ploidies option? For example, are triploid_homo or tetraploid_hetero appropriate input values?

**Response:** We are sorry for this confusion. The argument ‘--simulation_ploidies’ can include any combination of ‘haploid’, ‘diploid_homo’, ‘diploid_hetero’ and ‘ref:<nref>_var:<nvar>’ (where <nref> / <nvar> are the number of reference / alternative chromosomal copies, respectively). For example, setting ‘--simulation_ploidies ref:3_var:1’ simulates reads assuming a tetraploid genome, where three chromosomes are like the reference and one has the SVs. We have rewritten the methods and the help section (of this module) to clarify which options are available.

Perhaps it would be easier with just a number for the ploidy (as with the call_CNV module although I see it is limited to 1 or 2?) and an option for zygosity (homo/hetero) so that all combinations are possible?

**Response:** We think that setting different arguments for ploidy and zygosity may not be as straightforward as the reviewer is proposing, since some users may want to simulate more complex zygosities (for example a tetraploid where 1/4 of the chromosomes have variants). We believe that the new clarified methods and help section are sufficient to allow a proper customization of the simulation ploidies and zygosities.

Regarding the ‘call_CNVs’ module, the ploidy argument only has an effect on the algorithm CONY, which currently accepts only ploidies 1 and 2. It is possible to run ‘call_CNVs’ on genomes with other ploidies by skipping CONY (and using the other algorithms where ploidy is not considered). We agree that this was unclear, and we have clarified it in the help message from the ‘call_CNVs’ module.

6. Page 7 line 38, you mention how the optimisation is in order to call all SV types. Have you tried splitting it in order to find the optimum parameters per SV type? And if so, does it help much?

If this was added, it could perhaps be indicated to be a 'slow but accurate' option (assuming it is more accurate).

**Response:** This is a very interesting point. We designed the parameter optimization module assuming that, because the optimal parameters are chosen to be accurate across all types of simulated genomes and SVs (global parameters), the accuracy for each SV is maximized. However, we did not show any data that validates this assumption, and we agree that using specific parameters for each SV type (SV type-specific parameters) may be necessary to maximize accuracy. To test whether using such SV type-specific parameters offers any benefit we used the accuracy measurements from our testing on different species and types of simulations (Figure 3). We find that the accuracy yielded by global and SV type-specific parameters is almost identical (see the new Figure S4), which validates our approach of running SV calling once (with a single set of global parameters) for each sample. These findings have been added to the revised manuscript.

7. The modules to find repetitive regions and use this information during SV simulation is a very nice addition. It is also great to see how the impaired results of whilst using this step during simulation could perhaps explain the discrepancy between simulated and real datasets, in that SVs are commonly found in repetitive regions.

**Response:** We thank the reviewer for these positive remarks.

8. Testing across samples and species nicely shows how each genome can require a very different set of parameters. Could you perhaps give some details as to which parameters are most variable among different species and if these parameters/differences may say something about the genomic background? This could also be done for test with and without the consideration of homologous regions/using real data. This would perhaps only be interesting if few parameters contained most of the variability.

**Response:** We agree that this is a relevant question, insufficiently addressed in the manuscript. To further understand why certain parameter choices impact SV calling accuracy we analyzed how changing each parameter in isolation (keeping all other parameters default) affects accuracy in different species and types of simulations. These results are shown in the new Figure S5B and we believe that they provide significant insights that address the question raised by the reviewer. In general, we find that the accuracy reached by changing single parameters is mostly below the accuracy reached from combinatorial parameter optimization. This suggests that there is often a complex interplay between different parameters, which justifies the need for such combinatorial optimization. However, this analysis also revealed parameters that have large effects: the set of vcf ‘FILTER’ tags defining accepted breakpoints, the coverage thresholds that define tandem duplications and deletions and the minimum number of supporting reads per variant.

We also find that, as predicted by the reviewer, these parameters have particular effects in some species and types of simulations. For example, requiring *de novo* assembly support for SVs in *C. albicans* and *C. glabrata* species is too stringent, which may reflect unique genomic features or technical properties in the *Candida* samples driving worse assembly performance. On another line, diploid species require particular (less stringent) coverage filters, likely to find heterozygous deletions and tandem duplications. Finally, we see that filtering out variants that overlap repetitive elements can affect accuracy mostly in realistic simulations, likely due to the fact that real variants could appear around such repeats. Conversely, the minimum number of supporting reads per variant had sample-specific (not species-specific nor simulation-specific), which we attribute to varying coverage, read lengths or sequencing quality. We now report all the findings in the manuscript.

9. Page 9 line 46, How exactly is the redundancy defined between calls from the 'call_SV' and 'call_CNV' modules?

**Response:** We agree that this is not clear. To remove redundancy, the module skips any CNV called by ‘call_CNVs’ that overlaps reciprocally (by at least an 80% of the region) a CNV called by ‘call_SVs’ using *bedmap* from the *bedops* tool. This has been clarified in the methods.

10. 1Page 12 line 40, did you forget to mention the use of the 'call_CNV' module?

**Response:** We did not test the call_CNVs module on all the datasets. We designed the CNV calling module as a complement to perSVade, allowing for the identification of several types of variants in the same framework. However, this module is just a (standard) implementation of available software, without much scientific novelty. This is why this work is centered on the (actually novel) SV calling features of perSVade, which are unrelated to the ‘call_CNVs’ module.

11. Could you add a 'fast' option to speed up the process for large genomes? What process takes up the most of the time? Could the parameter optimisation steps be run on just half or a subset of the chromosomes (as performed for the human genomes, Page 13 line 7)?

**Response:** This is a very good point. There are several options to speed up the process of parameter optimization and SV calling. First, users may rationally design the parameters (based on parameters optimized for similar samples and/or the benchmarking shown in this work) instead of inferring them with the ‘optimize_parameters’ module (the most computationally intensive part (see Figure S2)). Second, marking duplicates in read alignment may be skipped, which can be very costly for large datasets. Third, the ‘optimize_parameters’ currently includes an option (as noted by the reviewer) to perform simulations in a subset of the chromosomes (*--simulation_chromosomes*). We agree that these recommendations were missing, and they are now clarified in the methods and discussion.

12. Is there some idea as to why there is little improvement in the detection of SVs in C. neoformans?

**Response:** We agree that this is a relevant concern, insufficiently addressed in the manuscript. We found the answer to this question by investigating the difference in parameter optimization between *C. neoformans* and *C. glabrata*. We consider that this difference is unlikely driven by intrinsic genomic differences between the two species, as both have small (<20Mb) haploid genomes with low content of simple repeats or low complexity regions. We hypothesized that *C. glabrata* samples have an excessively high coverage (>300x, while *C. neoformans* samples have a ~30x coverage) which may constrain SV calling accuracy and require optimized parameters. To test this, we measured the accuracy of different parameter sets on the *C. glabrata* simulations with randomly downsampled coverages. As hypothesized, we find that most parameters are accurate on the *C. glabrata* with 30x coverage, while simulations with lower (10x) and higher (100x-500x) coverage require specific parameters (see the new Figure S7). These results suggest that 30x could be the optimal coverage for perSVade, which is reasonable given that gridss was developed for human datasets with similar coverage. In summary, the coverage of *C. neoformans* samples was optimal for SV calling, so that the filtering strategy is probably not so relevant, and even the default parameters work well for these samples. We now include these findings in the manuscript. In addition, we now mention that the differences between species can be both due to intrinsic genomic properties and/or technical variation (i.e. coverage). Note that we chose these datasets to reflect real samples for each species, which can have very different coverages.

Is there any example where it gets worse and in the case that the defaults are the optimum can it make no changes to the parameters?

**Response:** We do find parameter sets that are less accurate than the default in *C. neoformans*, indicating that not all parameter combinations are optimal in this species. For example, we find that filtering out SVs overlapping repeats yields lower accuracy on the realistic simulations (Figure S5B). This is likely due to the fact that many real SVs happen around such repeats (i.e. SVs related to transposable elements). This means that, although SV filtering can be more flexible in *C. neoformans*, there are still some parameter choices that can reduce accuracy.

13. Using the tool requires the use of many different modules each with their own output which in turn needs to be fed into the next module. Would you be able to provide an option in which the tool does it all in one go (trimming, aligning, optimizing, calling SVs/CNVs, merging, etc)? It seems there was already this function previously but you are removing this feature?

**Response:** We agree that this seems a reasonable option, and we actually initially developed perSVade as a one-liner wrapper script. However, as we added modules to the pipeline this structure got more complicated to maintain and use. In fact, many of the errors and bugs found by the earlier users of perSVade were related to the lack of modularity. This is why we present a modular structure as a final version. The one-liner wrapper is still available at <https://github.com/Gabaldonlab/perSVade/blob/master/scripts/perSVade.py>, but it has an overwhelmingly high number of arguments (68 in total) to run all the modules. It may seem that having a one-liner version improves usability, but our experience advising many beta-testers is completely opposite. It is very difficult to properly use this one-liner pipeline, because the interactions between so many arguments are not always easy to predict for the user, generating a lot of confusion. In addition, a modular version improves customization and understanding of what the pipeline is doing. This is why we decided to drop support for this one-liner version. We think that the modular structure is more user friendly, and we consider that adding a one-liner version can complicate usage. We have clarified this in the data availability statement.

14. The FAQs page on the github is rather detailed and clarifies simple questions that arise during the process of running the modules.

**Response:** We thank the reviewer for this positive appreciation.

**Second round of review**

**Reviewer 1**

The authors have satisfactorily addressed my questions.

**Reviewer 2**

I find myself, upon this second round of review, further convinced of the tool's utility especially now as the manuscript comments upon the complexity involved in parameter tuning. I have only a few more comments to make.

1. Personally, the authors' choice of using gridss as the short-read SV calling tool and the large amount of work required in order to create this parameter optimising tool, on top of gridss, has been clearly and convincingly explained and clarified. However, a comparison of perSVade to another tool, for example Manta, would help illuminate what sort of relative improvement perSVade is bringing and potentially further encourage users to move towards using perSVade. The human dataset, as mentioned has already used for benchmarking in Cameron et al., serves as a perfect test-set. However, it is understandable how this may be outside of the scope of this manuscript and that self-performed benchmarking can be fraught with bias. I leave this only as a comment with no specific request for any benchmarking to be performed.

2. Page15 line23, misspelling of perSVade

3. In figure 2 and s3 the position of the default and optimised points change from left to right and vice versa without any apparent reason to do so. It is just a minor inconvenience when looking at the plots.

4. In the new analysis using SV type-specific vs global parameters it is interesting that it appears that the global parameters on occasion perform better than that of the SV-specific, particularly for tandem duplications. Does that suggest that tandem duplications should perhaps not be used during parameter training?

5. Due to the results of your coverage analysis, should ~30X therefore be recommended for perSVade and an option for down-filtering be offered/set as default? This could also potentially improve the speed in datasets with excessive coverage and large genomes.

**Authors Response**

**Point-by-point responses to the reviewers’ comments:**

Reviewer #2: I find myself, upon this second round of review, further convinced of the tool's utility especially now as the manuscript comments upon the complexity involved in parameter tuning. I have only a few more comments to make.

1. Personally, the authors' choice of using gridss as the short-read SV calling tool and the large amount of work required in order to create this parameter optimising tool, on top of gridss, has been clearly and convincingly explained and clarified. However, a comparison of perSVade to another tool, for example Manta, would help illuminate what sort of relative improvement perSVade is bringing and potentially further encourage users to move towards using perSVade. The human dataset, as mentioned has already used for benchmarking in Cameron et al., serves as a perfect test-set. However, it is understandable how this may be outside of the scope of this manuscript and that self-performed benchmarking can be fraught with bias. I leave this only as a comment with no specific request for any benchmarking to be performed.

**Response:** We thank the reviewer for the positive comments. We agree that this comparison to Manta could be very interesting, but we have decided to leave to further research to ensure that this manuscript is concise enough.

2. Page15 line23, misspelling of perSVade

**Response:**This has been changed, thanks.

3. In figure 2 and s3 the position of the default and optimised points change from left to right and vice versa without any apparent reason to do so. It is just a minor inconvenience when looking at the plots.

**Response:** This is a good point. We noticed that the dataframe objects used for plotting these figures were sorted by species and type of simulation, which generates these differences in the ordering of the parameters in different panels. However, within each panel the ordering is consistent, and we think that this is sufficient to represent the large differences in accuracy seen between default and optimized parameters.

4. In the new analysis using SV type-specific vs global parameters it is interesting that it appears that the global parameters on occasion perform better than that of the SV-specific, particularly for tandem duplications. Does that suggest that tandem duplications should perhaps not be used during parameter training?

**Response:** This is a good point, and it can be explained from the behavior of the parameter *min_rel_coverage_to_consider_dup* (the minimum relative coverage required for a tandem duplication (TDUP) - like breakpoint to be classified as an actual TDUP). When choosing TDUP-specific parameters in diploids (which are the species where the global parameters sometimes perform better), the ‘optimize_parameters’ module tries a range of values between 0 and 3.5. However, the parameter optimization for other SV types skip changing this parameter (as it only affects TDUPs) and set *min_rel_coverage_to_consider_dup* to 1.3 (which is a rationally designed value). This exact 1.3 value is not considered in the range of values of the TDUP-specific parameter optimization (to avoid redundancy in the evaluated values and speed up the process). In some cases, 1.3 is actually the optimal value, so that parameters optimized for other SV types perform better than any of the TDUP-specific parameters. This explains why sometimes the global parameters (which are optimized specifically for one SV type, but are also the ones performing best in all the other SV types and simulations) perform better than the TDUP-specific parameters. We chose this design for the parameter optimization to reduce the total number of parameter values evaluated and speed up the process.

However, we observe that this is not a universal behavior, since other thresholds can be optimal in some samples, suggesting that tandem duplications should be considered in parameter optimization. We have clarified this in the figure legend.

5. Due to the results of your coverage analysis, should ~30X therefore be recommended for perSVade and an option for down-filtering be offered/set as default? This could also potentially improve the speed in datasets with excessive coverage and large genomes.

**Response:** This is an interesting idea, but we consider that setting a downsampling step as default may be risky in some cases. Note that our testing is limited to some samples in a few species (which have particular genome features, insert sizes and read lengths), and we cannot guarantee that this 30x coverage will be universally optimal. We consider that setting an automatic downsampling may yield inaccurate results on other species or types of samples, where higher coverage could be necessary. We agree that downsampling may be a good strategy to improve both accuracy and performance, and we now discuss this in the revised manuscript. However, we believe that this should be an option and not a default behavior.
